# Decoding the genomic repertoire of fosfomycin resistance genes in staphylococci

**DOI:** 10.1038/s41467-026-77511-2

**Published:** 2026-09-10

**Authors:** Yiyi Chen, Feiteng Zhu, Mengke Ye, Yueqin Hong, Peiqi Wang, Haiping Wang, Zhengan Wang, Xiaoxing Du, Lu Sun, Yunsong Yu, Yan Chen

**Affiliations:** 1 Department of Pathology, Sir Run Run Shaw Hospital, Zhejiang University School of Medicine, Hangzhou, China; 2 Key Laboratory of Microbial Technology and Bioinformatics of Zhejiang Province, Hangzhou, China; 3 Department of Infectious Diseases, Sir Run Run Shaw Hospital, Zhejiang University School of Medicine, Hangzhou, China; 4 Regional Medical Center for National Institute of Respiratory Diseases, Sir Run Run Shaw Hospital, Zhejiang University School of Medicine, Hangzhou, China

**Keywords:** Molecular medicine, Antimicrobial resistance

## Abstract

Fosfomycin is regaining clinical relevance for multidrug-resistant staphylococcal infections, yet the diversity, evolutionary origin and nomenclature of staphylococcal fosfomycin resistance genes remain inconsistently defined. We surveyed 17,024 publicly available genomes representing 14 clinically common *Staphylococcus* species to curate a catalogue of fosfomycin-modifying *fos* homologues. By integrating amino-acid identity, phylogenetic topology, genomic context and population-level distribution, we established a reproducible classification framework that resolved species- or lineage-associated intrinsic genes, including *fosSA*, *fosSE*, *fosSC*, *fosSCp* and *fosSL*, from acquired determinants, including *fosB*, *fosD*, *fosY* and ten prophage-associated families designated *fosSΦA–J*. Intrinsic genes showed conserved local contexts, whereas prophage-borne determinants occupied diverse insertion sites, supporting distinct evolutionary trajectories. Functional assays showed variable effects on fosfomycin susceptibility. Deleting *fosSA* in MRSA reduced the fosfomycin MIC 16-fold, and complementation restored resistance. This study provides a unified nomenclature and evolutionary framework for staphylococcal *fos* genes and highlights the need to monitor both lineage-associated and mobile *fos* determinants.

## Abstract (web summary)

Here, the authors use 17,000 public genomes to systematically classify and unify nomenclature for fosfomycin resistance genes in *Staphylococcus*.

## Introduction

Antimicrobial resistance is rapidly emerging on a global scale and has become one of the most serious threats to public health^1^. Even resistance to last-line antibiotics such as daptomycin, linezolid and vancomycin has been reported in methicillin-resistant *Staphylococcus aureus* (MRSA)^2^. The stagnation in antibiotic development has renewed interest in older antibiotics, such as fosfomycin. Fosfomycin has been evaluated as an adjunctive or combination option for severe *S. aureus* infections, including bacteraemia and endocarditis^3,4^. Recent randomised trials and pooled analyses suggest that its clinical value may depend on infection complexity, patient subphenotype and treatment context, rather than being uniform across all patients^5^. This continued clinical interest highlights the need to better understand the genetic basis of fosfomycin resistance in staphylococci.

Fosfomycin, which interferes with the first cytoplasmic step of bacterial cell wall biosynthesis by specifically inhibiting the enzyme UDP-N-acetylglucosamine-3-enolpyruvyltransferase, possesses broad-spectrum activity against both Gram-positive and Gram-negative bacteria^6^. In *Staphylococcus*, fosfomycin resistance frequently arises from missense or frameshift mutations in the *glpT* and *uhpT* transporters that abolish fosfomycin uptake, as previously reported^6,7^. Additionally, fosfomycin-modifying enzyme genes such as *fosB, fosD* and *fosY*^8–11^ located on plasmids or genetic islands, have been identified in staphylococci, although their prevalence remains low. These genes encode bacillithiol transferases that confer resistance by enzymatic inactivation of fosfomycin. The presence and potential dissemination of these acquired *fos* genes underscore the need for accurate annotation and surveillance of fosfomycin resistance determinants in staphylococci.

Previous studies have shown that a series of chromosomal *fosA* homologues are widespread among Gram-negative bacteria, with amino acid identities of 62–80% among these homologues^12^. Whether similar situations occur in Gram-positive bacteria, however, remains largely unexplored. Importantly, in both Gram-negative and Gram-positive bacteria, the function, nomenclature and classification of chromosomally encoded *fos* genes have not been well defined. In earlier work, we identified *fos*-family genes as *fosY* in *S. aureus*^11^ and *fosSC* in *Staphylococcus capitis*^13^. More recently, we identified *fosSA*, a gene that shares 72% amino acid identity with plasmid-borne *fosB* and is widely distributed among *S. aureus* strains^14^. However, chromosomal *fosSA* and related *fosB*-like homologues have often been grouped under broad *fosB* annotations in public databases or previous studies^15,16^, despite being distinct from acquired plasmid-borne *fosB*. This inconsistent annotation has contributed to ambiguity in the taxonomy and surveillance of *fos* genes in staphylococci.

Despite these findings, a comprehensive overview of *fos* gene diversity, distribution and functional contribution to resistance in *Staphylococcus* species is lacking. This study aimed to systematically identify and classify *fos* genes across publicly available staphylococcal genomes, to identify *fos* variants not represented in current nomenclature, and to assess their functional roles in fosfomycin resistance. We also propose a revised nomenclature and classification framework for *fos* genes in staphylococci, based on sequence similarity, genomic context and evolutionary relatedness.

## Results

### Diverse *fos* families were identified in staphylococci

By applying our classification framework to 17,024 publicly available genomes from 14 clinically common *Staphylococcus* species, we identified a broad repertoire of *fos*-like genes and compiled a final sequence dataset comprising 151 *fos*-family sequences. These included previously reported *fosSA*, *fosSC*, *fosB*, *fosD* and *fosY* genes and their allelic variants, as well as 13 additional *fos* gene types that we designated *fosSE*, *fosSCp*, *fosSL* and the prophage-associated families *fosSΦA–J*. Together, these findings indicate that the *fos* repertoire in staphylococci is broader than reflected in current annotations.

**Fig. 1:**
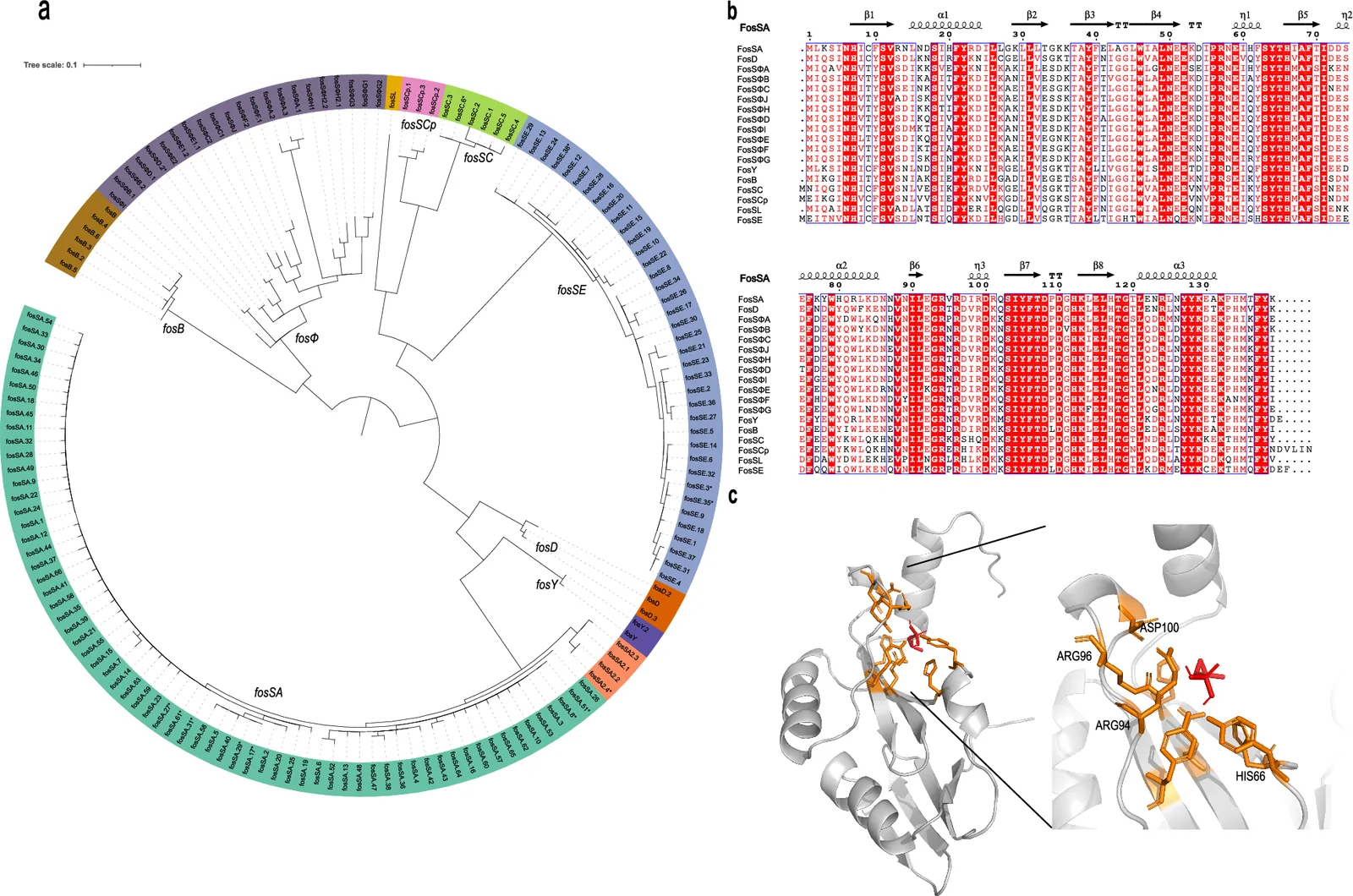
Evolutionary and structural characterisation of Fos families in staphylococci. **a** Maximum-likelihood phylogeny of representative Fos amino-acid sequences. Colours denote major *fos* gene families; prophage-associated *fosSΦA–J* families are grouped as *fosSΦ*. Asterisks indicate alleles containing premature stop codons. Scale bar, amino-acid substitutions per site. **b** Multiple sequence alignment of representative Fos proteins showing conserved residues and predicted secondary-structure elements. **c** AlphaFold-predicted FosSA structure with docked fosfomycin. Representative conserved pocket residues are shown as orange sticks and fosfomycin as red sticks. The close-up view highlights the predicted substrate-binding pocket.

The representative *fos* family phylogeny showed that the major *fos* categories formed distinct evolutionary lineages, supporting their classification as separate gene families (Fig. 1a). The prophage-associated *fosSΦA–J* genes clustered with *fosB*-like sequences but formed a separate group from the intrinsic chromosomal families, consistent with their distinct prophage-associated genomic contexts. Focused phylogenetic and amino-acid identity analyses further resolved the prophage-associated genes into individual *fosSΦ* families, including the separation of closely related but phylogenetically unsupported borderline groups into independent lineages (Supplementary Fig. 1A). Pairwise amino-acid identity analysis of representative *fos* families showed substantial sequence divergence, with identities ranging from approximately 59.7–91.4% among major families (Supplementary Fig. 1B).

Despite this sequence diversity, representative Fos proteins retained the characteristic β-α-β-β-β architecture of the vicinal oxygen chelate (VOC) enzyme superfamily and conserved residues around the predicted substrate-binding region (Fig. 1b). Mapping of representative conserved pocket residues onto the AlphaFold-predicted FosSA structure with docked fosfomycin further supported the presence of a conserved substrate-accommodation framework across Fos proteins (Fig. 1c).

### Classification and nomenclature of *fos* genes

Based on the classification framework, *fos* genes were categorised as intrinsic or acquired according to prevalence, distribution pattern, local genomic context and association with mobile genetic elements. The intrinsic group included *fosSA*, *fosSC*, *fosSE*, *fosSL* and *fosSCp*, which were associated with specific *Staphylococcus* species or major lineages and generally showed conserved local genomic contexts. Their names were assigned by combining *fos* with the species abbreviation. In contrast, the acquired group comprised previously reported *fosB*, *fosD* and *fosY*, for which established names were retained.

Notably, several *fos* gene families identified in this study were embedded within staphylococcal prophage islands. Among them, the *fosSΦA–J* variants formed a distinct phylogenetic cluster and were designated with the phage symbol ‘Φ’ to reflect their typical genomic context, with different letters (A–J) used to distinguish individual gene families. Importantly, prophage association alone was not considered indicative of mobility, and intrinsic or acquired status was assigned based on evolutionary behaviour rather than genomic annotation.

To facilitate comparison with previous literature and public database annotations, we compiled a nomenclature cross-reference table summarising previously used names or database annotations, representative accessions or locus identifiers, and the corresponding names proposed or retained in this study (Supplementary Data 1).

### Intrinsic *fos* genes were widespread in staphylococci

#### *S. aureus* and *S. argenteus*

**Fig. 2:**
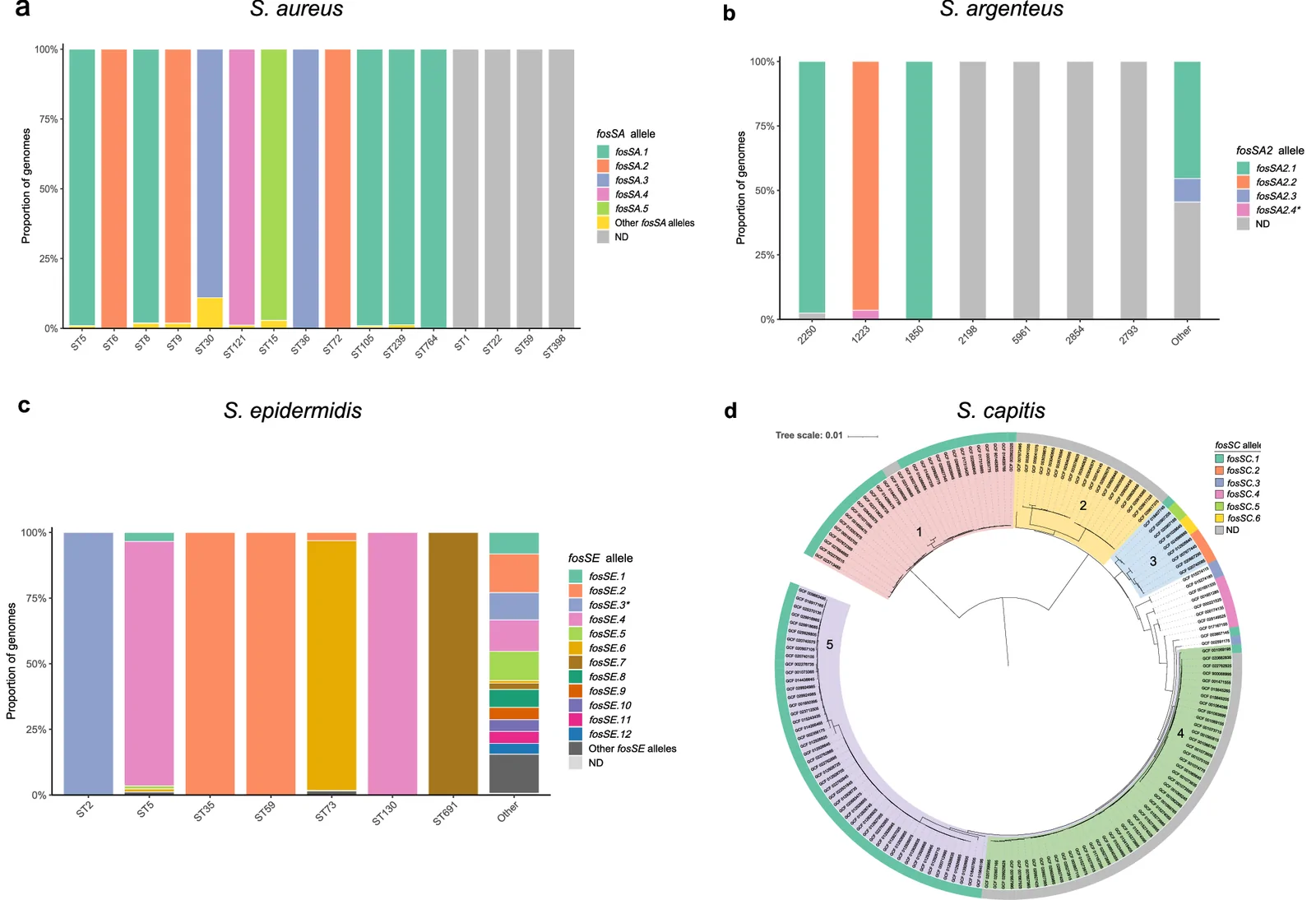
Clone- and clade-associated distribution of intrinsic *fos* alleles in representative *Staphylococcus* species. For *S. aureus* (**a**), *S. argenteus* (**b**) and *S. epidermidis* (**c**), intrinsic *fos* allele distributions are shown across major sequence types using stacked bar plots. For *S. capitis* (**d**), *fosSC* distribution is shown on a core-genome phylogeny. Colours indicate allele categories within each panel. ND indicates no corresponding intrinsic *fos* allele detected. Asterisks denote alleles containing premature stop codons. Source data are provided as a Source Data file.

We identified 66 *fosSA* alleles in *S. aureus*, with amino acid sequence identities ranging from 94.96% to 99.28%. In the dataset, *fosSA* was detected in 9328/14,281 *S. aureus* genomes (65.32%). Rather than showing uniform species-wide distribution, *fosSA* displayed a marked ST-associated pattern involving both presence/absence and allele composition. Several major STs, including ST5, ST8, ST105, ST239 and ST764, were dominated by *fosSA.1*, whereas ST6, ST9 and ST72 mainly carried *fosSA.2*. In contrast, several major STs, including ST1, ST22, ST59 and ST398, lacked detectable *fosSA* in the analysed genomes (Fig. 2a). This pattern included both hospital-associated epidemic lineages, such as ST5 and ST239, and community-associated lineages, such as ST8, among *fosSA*-positive STs, whereas other community-associated or livestock-associated lineages, including ST1, ST59 and ST398, lacked detectable *fosSA*.

Notably, we also identified *fosSA* homologues in several *S. argenteus* clones. These sequences shared ≥92% amino acid identity with *fosSA* in *S. aureus*, and were therefore designated as *fosSA2*, reflecting their evolutionary relatedness within the *fosSA* gene family. In *S. argenteus*, *fosSA2* was detected in 158/219 genomes (72.15%) and showed ST-associated presence/absence and allele distribution. ST2250 was dominated by *fosSA2.1*, whereas ST1223 was dominated by *fosSA2.2*. In contrast, several STs, including ST2198, ST5961, ST2854 and ST2793, lacked detectable *fosSA2* in the analysed genomes (Fig. 2b).

#### *S. epidermidis*

For *S. epidermidis,* 38 *fosSE* alleles were identified, with the amino acid sequences varying between 92.25% and 99.30%. In the dataset, *fosSE* was detected in 1,156/1,160 genomes (99.66%), indicating near species-wide carriage. Despite this high prevalence, *fosSE* showed strong ST-associated allele distribution (Fig. 2c).

#### *S. capitis*

In *S. capitis*, *fosSC* was detected in 95/162 genomes (58.64%), with the amino acid sequences varying between 97.86% and 99.29%. The distribution of *fosSC* was structured by phylogenetic background. Several clades were enriched for *fosSC*, whereas others lacked detectable *fosSC* (Fig. 2d).

#### *S. caprae*

Similarly, *fosSCp* was detected in 14/20 *S. caprae* genomes (70.00%), with amino acid sequence identities ranging from 97.93 to 98.62%. The distribution of *fosSCp* was associated with phylogenetic background, with *fosSCp.1* concentrated within a major clade and *fosSCp.3* present in several additional branches (Supplementary Fig. 2). This distribution pattern should be interpreted in the context of the limited number of available *S. caprae* genomes.

#### *S. lugdunensis*

In *S. lugdunensis*, *fosSL* was detected in 98/101 genomes (97.03%), indicating near species-wide carriage. The currently identified *fosSL* sequences were highly conserved, with limited allele-level variation.

### Genomic context of intrinsic *fos* genes

**Fig. 3:**
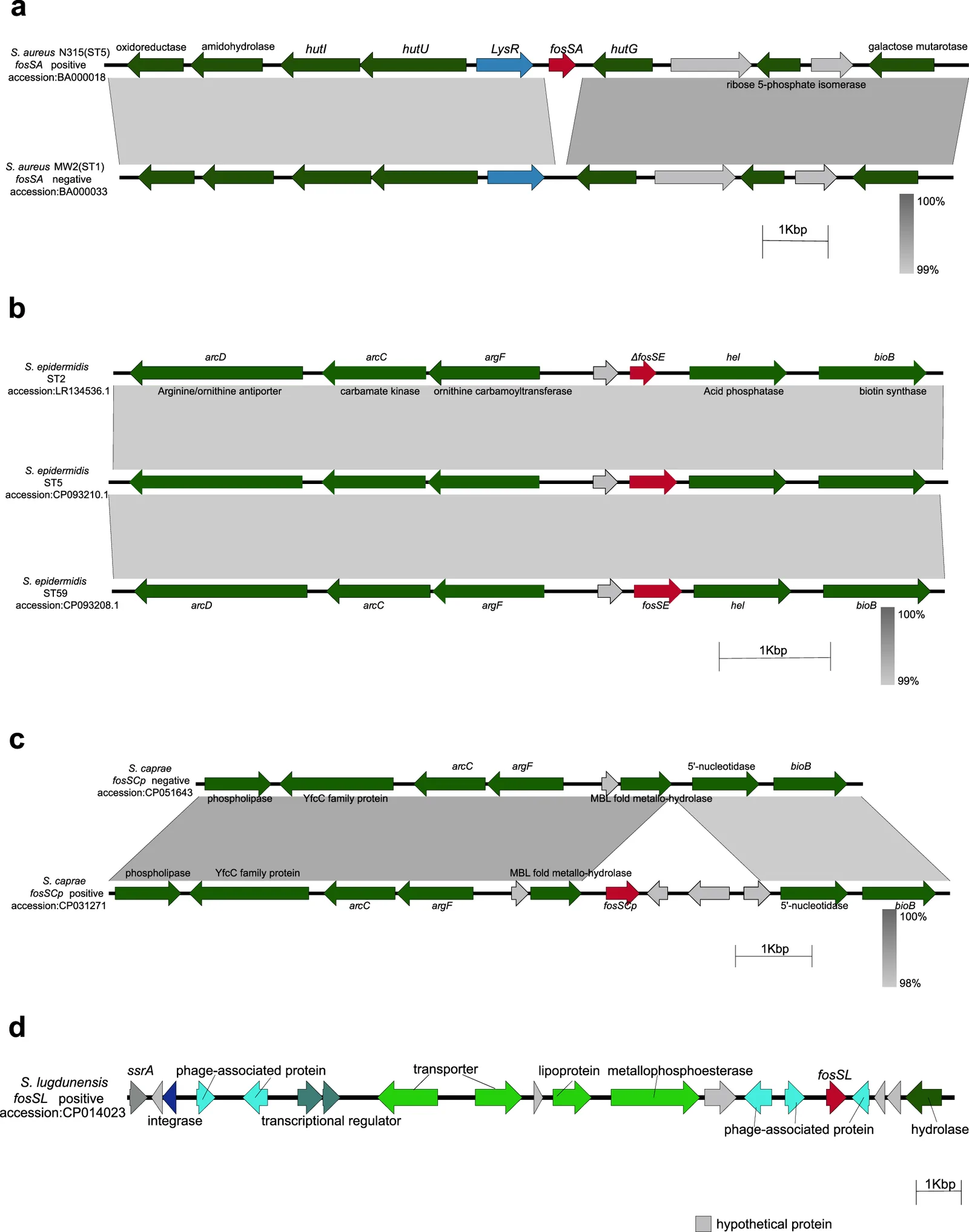
Genomic structures of intrinsic fos genes. **a** Genomic structure of *fosSA* in *Staphylococcus aureus* N315, with *fosSA* shown in red. **b** Representative genomic structures of *fosSE* from three major sequence types (STs), with *fosSE* highlighted in red. **c** Genomic structure of *fosSCp*, with *fosSCp* shown in red. **d** Genomic structure of *fosSL*, with *fosSL* shown in red.

*fosSA*-positive strain N315 (ST5) and *fosSA*-negative strain MW2 (ST1) were selected for comparison to examine the genomic surroundings of *fosSA*. The regions flanking *fosSA* were conserved, with no mobile elements detected (Fig. 3a). Notably, the upstream and downstream regions contained the *hutIUR* operon involved in histidine metabolism. Similarly, in *S. epidermidis*, the regions surrounding *fosSE* were conserved, with *agrF* and *hel* located upstream and downstream, respectively, both of which are associated with growth and metabolism (Fig. 3b). Likewise, no mobile elements were identified around *fosSCp*, and its flanking regions closely resembled those of *fosSE* (Fig. 3c). These observations indicate that most intrinsic *fos* genes examined here were located in conserved chromosomal contexts without obvious nearby mobility-associated genes. *fosSL* in *S. lugdunensis* represented a distinct case. Although it was located within a prophage-like region, *fosSL* was detected in nearly all analysed *S. lugdunensis* genomes and showed a conserved local genomic context (Fig. 3d). Therefore, within the genomes analysed, *fosSL* was classified operationally as an atypical lineage-associated intrinsic *fos* gene.

### Distribution and diversity of acquired *fos* genes

**Fig. 4:**
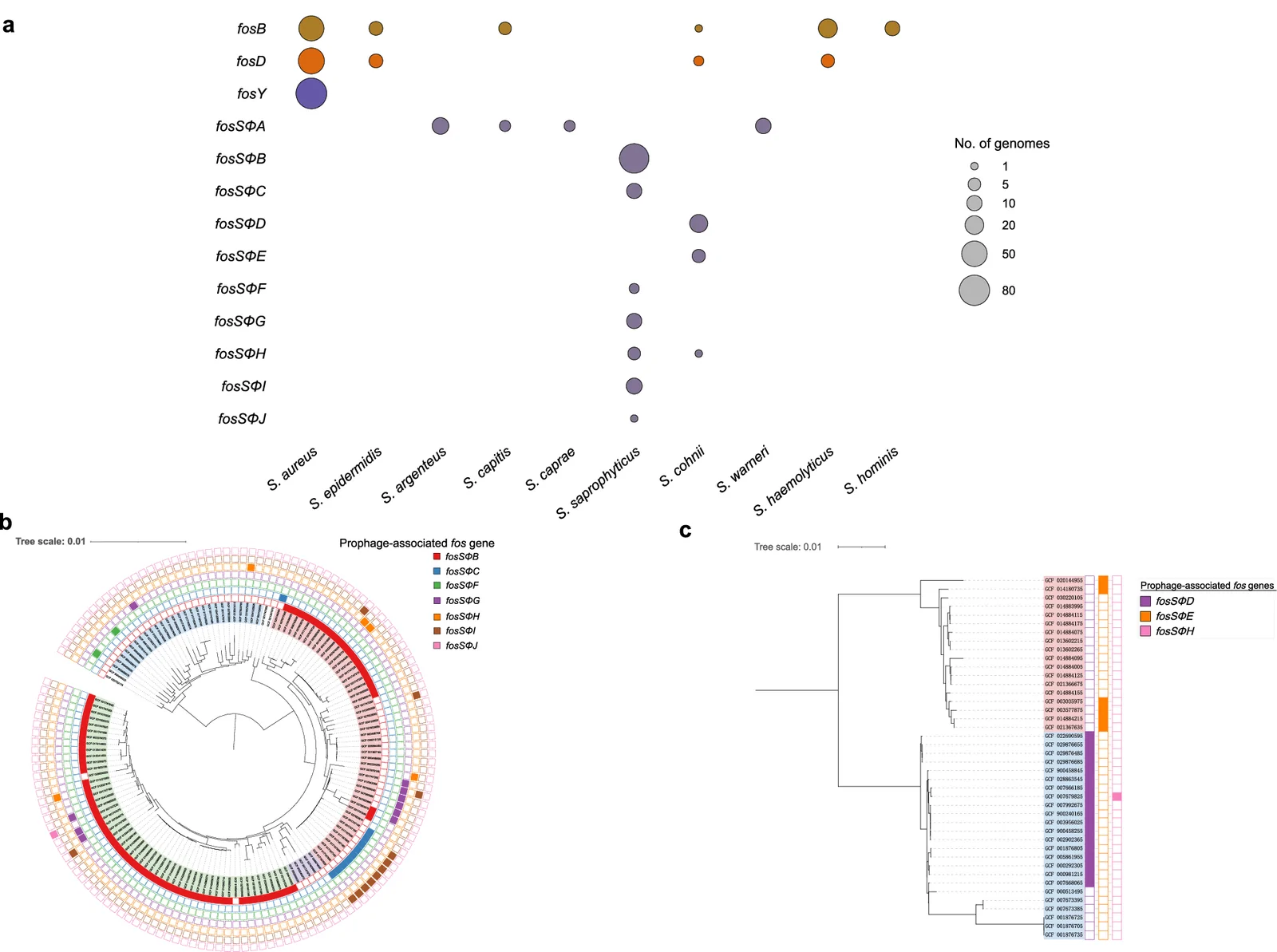
Distribution of acquired *fos*genes in staphylococci. **a** Bubble plot showing the distribution of acquired *fos* genes across *Staphylococcus* species. Bubble size indicates the number of genomes carrying each gene. Colours indicate different acquired *fos* categories, including plasmid-associated *fosB* and *fosD*, genomic-island-associated *fosY* and prophage-associated *fosSΦA–J* genes. **b** Core-genome phylogeny of *S. saprophyticus* showing the distribution of prophage-associated *fosSΦ* genes. Coloured tracks indicate the presence of different *fosSΦ* families. **c** Core-genome phylogeny of *S. cohnii* showing the distribution of prophage-associated *fosSΦ* genes. In (**b**, **c**), coloured blocks indicate the presence of the corresponding prophage-associated *fos* gene. Source data are provided as a Source Data file.

The plasmid-derived *fosB* gene cluster and its allelic variants, representing an early described *fos* family in staphylococci, exhibited broad but low-prevalence dissemination (<10% positivity rate) across multiple species including *S. aureus*, *S. epidermidis*, *S. capitis*, *S. haemolyticus*, *S. hominis* and *S. cohnii*, with a similar distribution pattern observed for plasmid-borne *fosD* alleles in *S. aureus*, *S. epidermidis*, *S. haemolyticus* and *S. cohnii* (Fig. 4a; Table 1). Notably, the *fosY* gene, located within a mobile genetic island flanked by transposase genes and insertion sequences, was exclusively detected in *S. aureus* (0.58% positivity rate) and showed evidence of clonal transmission among ST1, ST5 and ST8 lineages (Supplementary Data 7).

**Table 1:** Fosfomycin-resistance gene carriage among different *Staphylococcus* species

Species	Total no. of genomes	intrinsic *fos* genes	acquired *fos* genes	
			plasmid	prophage/genomic island
*S. aureus*	14,281	*fosSA* (9,328/14,281; 65.32%)	*fosB* (48/14,281; 0.34%), *fosD* (53/14,281; 0.37%)	*fosY* (83/14,281; 0.58%)
*S. epidermidis*	1160	*fosSE* (1156/1160; 99.66%)	*fosB* (7/1,160; 0.60%), *fosD* (7/1,160; 0.60%)	-
*S. argenteus*	219	*fosSA2* (158/219; 72.15%)	-	*fosSΦA* (14/219; 6.39%)
*S. capitis*	162	*fosSC* (95/162; 58.64%)	*fosB* (5/162; 3.09%)	*fosSΦA* (3/162; 1.85%)
*S. lugdunensis*	101	*fosSL* (98/101; 97.03%)	-	-
*S. caprae*	20	*fosSCp* (14/20; 70.00%)	-	*fosSΦA* (3/20; 15%)
*S. saprophyticus*	140	-	-	*fosSΦB* (74/140; 52.86%), *fosSΦC* (10/140; 7.14%), *fosSΦF* (2/140; 1.43%), *fosSΦG* (10/140; 7.14%), *fosSΦH* (5/140; 3.57%), *fosSΦI* (12/140; 8.57%), *fosSΦJ* (1/140; 0.71%)
*S. cohnii*	42	-	*fosB* (1/42; 2.38%), *fosD*(2/42; 4.76%)	*fosSΦD* (18/42; 42.86%), *fosSΦE* (6/42; 14.29%), *fosSΦH* (1/42; 2.38%)
*S. warneri*	131	-	*-*	*fosSΦA* (11/131; 8.40%)
*S. haemolyticus*	430	-	*fosB* (21/430; 4.88%), *fosD* (6/430; 1.40%)	-
*S. hominis*	125	-	*fosB* (9/125; 7.20%)	-
*S. chromogenes*	123	-	-	-
*S. simulans*	69	-	-	-
*S. carnosus*	21	-	-	-

Italicised text denotes bacterial species names and gene names, following standard nomenclature.

In addition to these elements, we identified a novel set of prophage-associated *fos* genes (*fosSΦA–J*) with distinct distribution profiles. Among them, *fosSΦA* was detected across the widest range of *Staphylococcus* species, including *S. argenteus*, *S. capitis*, *S. caprae* and *S. warneri*, suggesting broader interspecies dissemination than other prophage-associated *fosSΦ* families, with prevalence rates ranging from 1.85 to 15% (Table 1). This broad species distribution suggests that *fosSΦA* may have undergone interspecies dissemination through related prophage-associated elements.

By contrast, *fosSΦB–J* were largely restricted to coagulase-negative staphylococci. Specifically, *fosSΦB*, *fosSΦC*, *fosSΦF*, *fosSΦG*, *fosSΦI* and *fosSΦJ* were identified exclusively in *S. saprophyticus*, whereas *fosSΦH* was detected mainly in *S. saprophyticus* with one additional occurrence in *S. cohnii*. Among these *S. saprophyticus-*associated families*, fosSΦB* was the most prevalent (74/140, 52.86%) and clustered within defined phylogenetic lineages, while the others were sporadically distributed with prevalence below 10% (Fig. 4b). In *S. cohnii*, *fosSΦD*, *fosSΦE* and *fosSΦH* were detected at frequencies of 42.86%, 14.29% and 2.38%, respectively, with *fosSΦD*-positive isolates confined to a single clade, whereas *fosSΦE* was more broadly distributed across the phylogeny (Fig. 4c).

### Assessment of species-level sampling bias

Because public genome representation was uneven across *Staphylococcus* species, we further assessed whether sampling imbalance influenced the observed *fos* distribution patterns. Species-level subsampling analysis was performed by randomly selecting up to 120 genomes per species and repeating the procedure ten times. The major distribution patterns were retained across subsampled datasets, including the low prevalence of acquired *fos* genes in the highly sampled *S. aureus* population and the enrichment of prophage-associated *fosSΦ* genes in *S. saprophyticus* and *S. cohnii*. These results indicate that the main distribution patterns were robust to the subsampling scheme, although rare lineages and prevalence estimates should still be interpreted in the context of current public genome availability (Supplementary Data 2).

### Genomic context of prophage-associated *fos* genes

**Fig. 5:**
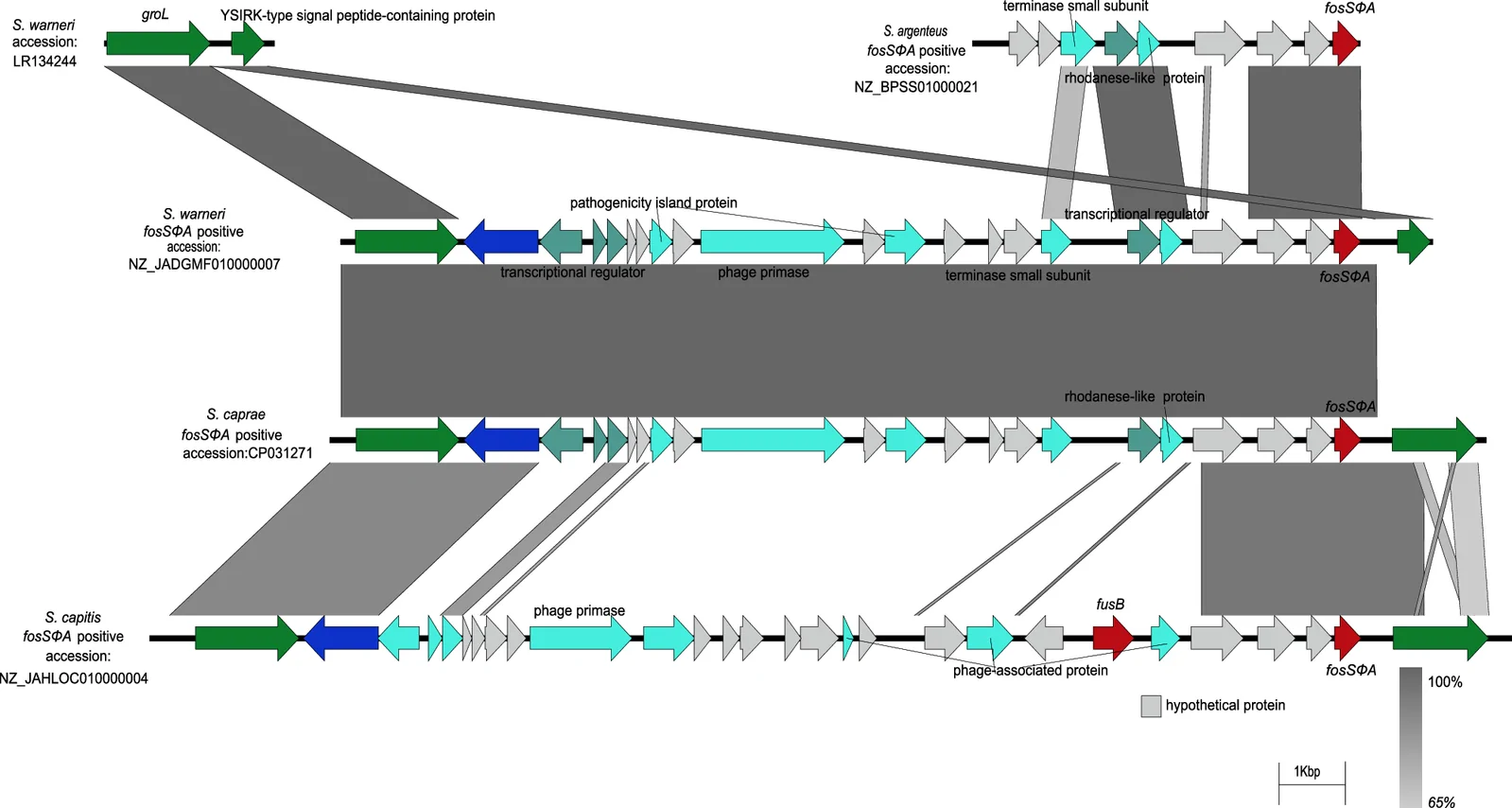
Genomic structures of *fosSΦA* in different *Staphylococcus* species. Genes of different functional categories are shown in different colours, with *fosSΦA* highlighted in red.

To investigate the mobility and potential origin of these *fos*-carrying prophages, we analysed their chromosomal integration sites and structural features. *fosSΦA*-positive prophages in *S. capitis, S. caprae* and *S. warner*i were consistently integrated downstream of the *groL* gene. Comparative genomic analysis revealed that the prophage structures in *S. caprae* and *S. warneri* were nearly identical. Although the prophage in *S. capitis* exhibited structural divergence, the integrase gene remained identical, suggesting a common origin. Similarly, the flanking region of *fosSΦA* in *S. argenteus* showed substantial similarity to that in *S. caprae* and *S. warneri*, indicating potential interspecies transmission (Fig. 5).

Other *fos*-associated prophages exhibited diverse but site-specific integration patterns. Prophages carrying *fosSΦB, fosSΦD*, *fosSΦE* and *fosSΦI* were inserted downstream of *ssrA*; *fosSΦC* was located near the *guaA* gene; *fosSΦF* was located near the *groL* gene and *fosSΦG* and *fosSΦH* were integrated adjacent to tRNA gene clusters. *fosSΦJ* was also embedded within a prophage-associated region containing mobility-related and pathogenicity-island-associated genes. Despite their structural variability, all prophages shared conserved functional modules encoding a site-specific integrase, primase, pathogenicity island proteins and terminase subunits (Supplementary Fig. 3).

### Functional contribution of representative *fos* genes to fosfomycin susceptibility

To assess the contribution of *fos* genes to fosfomycin resistance, we conducted heterologous expression experiments. Excluding the previously characterised *fosSC*^13^, intrinsic *fos* genes (*fosSA*, *fosSE*, *fosSCp*, *fosSL*) and a subset of acquired *fos* genes, including several prophage-derived representatives (*fosSΦA*, *fosSΦB*, *fosSΦC*, *fosSΦD*, *fosSΦE*) and the previously reported *fosD* and *fosY*, were cloned into the shuttle vector pTSSCm-pcap under the control of the vector-encoded promoter. Fosfomycin susceptibility testing showed that heterologous expression of representative *fos* genes increased fosfomycin MICs to different extents compared with the vector control (Table 2). Based on median MIC values from repeated measurements, *fosSE* and *fosSΦB* produced no detectable or only minimal MIC changes, whereas *fosSL* and *fosY* increased fosfomycin MICs to 16 mg/L. Higher MICs were observed for *fosD* (32 mg/L), *fosSA* and *fosSΦA* (64 mg/L), *fosSΦC* (128 mg/L), *fosSΦE* (256 mg/L), *fosSCp* (512 mg/L) and *fosSΦD* (1024 mg/L). These results indicate substantial functional heterogeneity among *fos* variants. In contrast, MICs for non-fosfomycin antibiotics, including cefoxitin, ceftobiprole, ciprofloxacin, gentamicin, rifampicin, linezolid, vancomycin and daptomycin, showed no consistent changes compared with the vector control.

**Table 2:** Antibiotic susceptibility of cloned, knockout and complemented strains

strain	MIC (mg/L)								
	FOS	CIP	GEN	RIF	LZD	VAN	DAP	FOX	CPT
RN4220/pTfosSA	64	0.12	0.25	0.008	2	1	0.25	1	0.25
RN4220/pTfosSE	0.5	0.12	0.25	0.008	2	1	0.25	2	0.25
RN4220/pTfosSCp	512 (256–512)	0.12	0.25	0.008	2	1	0.25	1	0.25
RN4220/pTfosSL	16	0.12	0.25	0.008	2	1	0.25	2	0.25
RN4220/pTfosD	32 (32–64)	0.12	0.25	0.008	2	1	0.25	1	0.25
RN4220/pTfosY	16	0.12	0.25	0.008	2	1	0.25	2	0.25
RN4220/pTfosSΦA	64	0.12	0.25	0.008	2	1	0.25	2	0.25
RN4220/pTfosSΦB	0.5 (0.5–1)	0.12	0.25	0.008	2	1	0.25	2	0.25
RN4220/pTfosSΦC	128 (128–256)	0.12	0.25	0.008	2	1	0.25	1	0.25
RN4220/pTfosSΦD	1024 (512–1024)	0.12	0.25	0.008	2	1	0.25	2	0.25
RN4220/pTfosSΦE	256	0.12	0.25	0.008	2	1	0.25	1	0.25
RN4220/pT	0.5 (0.25–0.5)	0.12	0.25	0.008	2	1	0.25	1	0.25
RN4220	0.5 (0.25–0.5)	0.12	0.25	0.008	2	1	0.25	2	0.25
LAC	16	8	<0.06	0.015	2	0.5	0.25	64	0.25
LACΔfosSA	1 (1–2)	8	<0.06	0.015	2	0.5	0.25	16	0.25
LACΔfosSA/pT	1	8	<0.06	0.015	2	0.5	0.25	16	0.25
LACΔfosSA/pT-fosSA	1024 (256–1024)	8	<0.06	0.015	2	0.5	0.25	32	0.25

Values are MICs in mg/L. Fosfomycin MICs were determined by agar dilution in repeated measurements and are reported as median values, with ranges shown in parentheses where variation was observed. Values without parentheses indicate identical results across repeated fosfomycin MIC measurements. Non-fosfomycin antibiotics were tested by broth microdilution.

*FOS* fosfomycin, *CIP* ciprofloxacin, *GEN* gentamicin, *RIF* rifampicin, *LZD* linezolid, *VAN* vancomycin, *DAP* daptomycin, *FOX* cefoxitin and *CPT* ceftobiprole.

To further validate the functional role of *fos* genes in clinical isolates, we generated a *fosSA* knockout in the MRSA strain LAC. The fosfomycin MIC for the Δ*fosSA* mutant decreased 16-fold compared to the wild-type strain (1 mg/L vs. 16 mg/L). Complementation with the empty vector did not affect the MIC, whereas complementation with *fosSA* restored resistance with an MIC of 1024 mg/L. A modest reduction in cefoxitin MIC was observed in the Δ*fosSA* mutant, but this change was not reversed by *fosSA* complementation and was comparable to the empty-vector control, suggesting it is independent of *fosSA* function. Susceptibility to other antibiotics, including ciprofloxacin, gentamicin, rifampicin, linezolid, vancomycin and daptomycin, was not altered (Table 2). Together, these findings confirm the critical role of *fosSA* in intrinsic fosfomycin resistance.

**Fig. 6:**
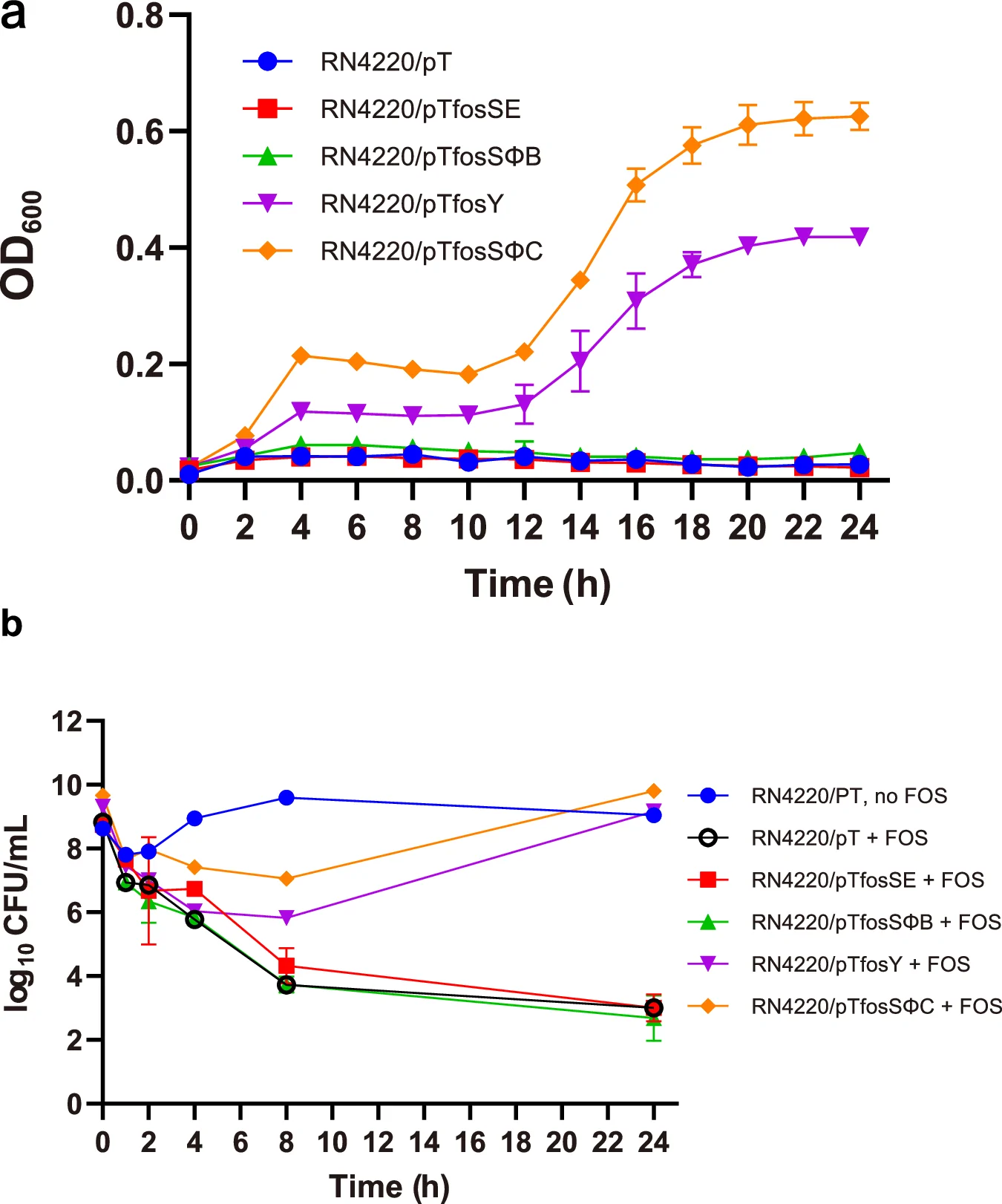
Growth and killing kinetics of representative *fos* variants under fosfomycin exposure. **a** Growth curves of *S. aureus* RN4220 carrying the empty vector or representative *fos* genes under fosfomycin (FOS) exposure at 16 mg/L. Bacterial growth was monitored by measuring OD_600_ over 24 h. OD_600_ values are shown at 2 h intervals for graphical clarity. **b** Time-kill assays of RN4220 carrying the empty vector or representative *fos* genes under FOS exposure. Viable bacterial counts were measured at the indicated time points and are shown as log_10_ CFU/mL. RN4220/pT without FOS was included as the untreated growth control, whereas RN4220/pT with FOS served as the FOS-exposed empty-vector control. All other groups were exposed to 16 mg/L FOS. Data are presented as mean ± SD from two independent biological replicates (*n* = 2), each started from an independent bacterial culture. No statistical comparisons are shown. Source data are provided as a Source Data file.

To further compare the functional behaviour of different *fos* variants beyond MIC measurements, we selected representative strains carrying low-, intermediate- and high-resistance variants (*fosSE*, *fosSΦB*, *fosY* and *fosSΦC)* and performed time-kill assays, tracking viable counts over time in the presence of fosfomycin (16 mg/L), complemented by growth curve analysis under the same conditions. The vector control showed a progressive reduction in viable counts, consistent with the bactericidal activity of fosfomycin. Strains expressing low-resistance variants, including *fosSE* and *fosSΦB*, displayed similar killing kinetics, with sustained decreases in bacterial counts and no evident recovery. In contrast, the strain expressing *fosY* exhibited an initial decline followed by partial regrowth at 24 h, whereas the *fosSΦC*-expressing strain maintained substantially higher viable counts throughout the assay and showed pronounced recovery at later time points (Fig. 6).

These results were consistent with the MIC phenotypes and indicate that different *fos* variants confer distinct capacities to sustain bacterial survival and subsequent growth recovery under fosfomycin pressure.

### Predictive structural analysis of representative Fos variants

To evaluate whether the variable fosfomycin resistance phenotypes could be explained by obvious structural differences, we performed AlphaFold Server-based structural prediction and fosfomycin docking using CB-Dock2. Representative Fos proteins were predicted to retain the conserved VOC scaffold, and most variants showed recurrent Tyr/His/Arg/Asp/Glu-associated residues around the predicted fosfomycin-accommodating region. Mapping representative conserved residues onto the predicted FosSA structure further supported conservation of the core substrate-accommodation framework (Fig. 1c).

However, predicted Vina scores did not show a simple linear relationship with fosfomycin MICs. Several variants with similar predicted binding energies displayed markedly different MIC phenotypes, whereas some high-resistance variants showed relatively weak predicted binding energies. In addition, most Fos variants retained recurrent pocket-associated residues despite large differences in resistance magnitude (Supplementary Data 3).

## Discussion

The *fos* gene family, encoding enzymes that inactivate fosfomycin, is broadly distributed in Gram-negative and Gram-positive bacteria^6^. However, the classification and nomenclature of *fos* genes have remained inconsistent across bacterial taxa because of sequence diversity, complex evolutionary relationships and overlapping historical names. Previous studies, particularly those focusing on Gram-negative bacteria, have provided an important foundation for distinguishing chromosomal and acquired *fos* determinants^12^. In staphylococci, however, a systematic classification framework has been lacking. Chromosomal, plasmid-associated, genomic-island-associated and prophage-associated homologues have often been annotated using overlapping *fosB*-like names^15,16^. In particular, the term *fosB* has frequently been applied broadly to diverse *fosB*-like homologues, including chromosomal species-associated genes that are evolutionarily and epidemiologically distinct from acquired plasmid-borne *fosB*^17^. This ambiguity complicates comparison across studies and obscures the distinction between lineage-associated intrinsic determinants and horizontally acquired resistance genes. Here, we systematically defined the *fos* repertoire across clinically relevant *Staphylococcus* species and established a reproducible classification and nomenclature framework integrating amino-acid identity, phylogenetic topology, genomic context and population-level distribution.

In this study, we developed an integrated workflow to identify and classify staphylococcal *fos* genes not represented in current classifications. Candidate homologues were detected by iterative sequence searches and then evaluated using amino-acid identity, phylogenetic placement, bootstrap support and genomic-context inspection. This strategy allowed us to resolve broad *fosB*-like annotations into distinct *fos* families and to distinguish species-associated intrinsic determinants from acquired genes located on plasmids, genomic islands or prophage-associated regions. This separation is important for resistance surveillance and genome annotation: intrinsic *fos* genes reflect lineage- or species-associated determinants that may shape baseline fosfomycin susceptibility, whereas acquired *fos* genes represent potentially mobile reservoirs that require monitoring for dissemination across lineages or species. Thus, the proposed classification framework provides a practical basis for comparing *fos* distribution among staphylococci, improving database annotation and assessing horizontal-transfer risk.

Fosfomycin is a long-established antibiotic that has regained clinical interest for the treatment of multidrug-resistant staphylococcal infections, particularly as part of combination therapy. Although recent clinical studies have evaluated fosfomycin-containing combinations for severe staphylococcal infections^5^, the genetic background underlying fosfomycin susceptibility in staphylococci remains incompletely understood. Our findings suggest that intrinsic *fos* genes may contribute to this background in a lineage-dependent manner. Deletion of *fosSA* in MRSA strain LAC reduced the fosfomycin MIC by 16-fold, and complementation restored resistance, confirming that *fosSA* contributes directly to intrinsic fosfomycin resistance. Importantly, the presence of an intrinsic *fos* gene does not necessarily mean that a strain is clinically resistant at baseline. Instead, these genes may be clinically overlooked because their contribution may appear as baseline MIC shifts or enhanced survival under fosfomycin pressure rather than overt high-level resistance in all genetic backgrounds. Such low- or intermediate-level protection could provide a permissive background for subsequent resistance evolution, especially when combined with transporter mutations affecting *glpT* or *uhpT*, which have been linked to high-level fosfomycin resistance in major *S. aureus* lineages such as ST5 and ST239^7^. Therefore, intrinsic *fos* genes should be considered as part of the genomic background shaping fosfomycin susceptibility and resistance evolution in clinically important staphylococci. Beyond their contribution to fosfomycin susceptibility, intrinsic *fos* genes may also have broader physiological roles. This possibility is suggested not only by previous functional observations for *fosSA*, but also by the evolutionary patterns observed in our dataset. Several intrinsic *fos* determinants showed lineage-associated or near-species-wide conservation and were embedded in conserved chromosomal contexts within the genomes analysed, a pattern more consistent with long-term maintenance in the core adaptive background than with purely opportunistic antibiotic resistance cargo. The chromosomal *fosSA* gene, previously often annotated as *fosB*, encodes a divalent-metal-dependent bacillithiol-S-transferase, and previous work showed that *fosSA* deletion reduced intracellular bacillithiol levels and impaired growth, suggesting links to redox homoeostasis and cellular fitness^18^. Similarly, the metal-dependent glutathione-S-transferase FosA7 has been linked to enhanced oxidative-stress tolerance in *Salmonella*^19^. In our genomic analysis, several intrinsic *fos* genes were located near genes involved in metabolism or stress-related processes, raising the possibility that at least some *fos* determinants contribute to broader adaptive networks beyond antibiotic detoxification. These observations suggest that *fos* genes in staphylococci may be best viewed not only as resistance determinants, but also as candidate physiological modulators whose native biological roles remain to be defined experimentally in the future.

A second clinically relevant finding is the diversity of prophage-associated *fosSΦ* genes in coagulase-negative staphylococci. CoNS are common colonisers but also important opportunistic and nosocomial pathogens^20^, and they have been recognised as reservoirs of genes that can contribute to the adaptation and resistance of more pathogenic staphylococci^21^. In particular, *S. saprophyticus* is a well-recognised cause of urinary tract infection, especially in young women^22^, making the discovery of diverse prophage-associated *fosSΦ* genes in this species clinically noteworthy. Although direct evidence linking these prophage-associated genes to treatment failure is not yet available, our cloning and susceptibility assays showed that representative *fosSΦ* genes can increase fosfomycin MICs to different extents. These findings suggest that prophage-associated *fos* genes in CoNS may represent an underappreciated reservoir of fosfomycin resistance determinants. From a clinical perspective, this highlights the need to include CoNS, not only *S. aureus*, in genomic surveillance of fosfomycin resistance, especially in settings where fosfomycin is used for urinary tract infections or as part of combination therapy.

The dissemination of acquired *fos* genes represents another important concern. Previous studies and our own data show that plasmid-associated *fosB* and *fosD* circulate among different *Staphylococcus* species^23,24^. In this study, we further identified prophage-associated *fosSΦ* genes as an additional mobile reservoir of fosfomycin resistance determinants. Among them, *fosSΦA* is particularly notable because it was detected in multiple *Staphylococcus* species, including *S. argenteus*, *S. capitis*, *S. caprae* and *S. warneri*. Comparative genomic analysis showed that *fosSΦA*-carrying regions from different species shared related prophage-associated architectures, including conserved phage modules and similar integration contexts. These genomic features support interspecies dissemination of *fosSΦA* through related prophage-associated elements. This finding extends the known mobile reservoir of *fos* genes from plasmids and genomic islands to prophage-associated elements, suggesting that bacteriophage-related regions may represent an underappreciated route for *fos* dissemination in staphylococci. From a One Health perspective^25^, whether prophage-associated *fos* genes circulate among animal-associated, environmental and clinical staphylococcal populations remains an important question for future genomic surveillance.

The functional analyses add an important layer to the genomic classification proposed in this study. Although the identified Fos variants retained conserved features of Fos-family fosfomycin-modifying enzymes, they were not functionally equivalent when expressed in the same *S. aureus* RN4220 background. Some variants produced little or no detectable MIC shift, whereas others conferred high-level fosfomycin resistance and supported bacterial survival and regrowth under fosfomycin exposure. This functional heterogeneity indicates that broad *fosB*-like annotation or the presence of conserved catalytic motifs alone is insufficient to predict resistance magnitude. Predictive structural analysis further supported this interpretation. Most Fos variants retained the conserved VOC scaffold and recurrent pocket-associated residues, but predicted docking scores did not correlate linearly with MICs. Thus, the heterogeneous resistance phenotypes observed in RN4220 cannot be explained solely by conserved catalytic residues or predicted fosfomycin binding energy. A more plausible model is that Fos-mediated resistance depends on additional factors, including productive substrate positioning, accommodation of the physiological thiol donor, catalytic turnover, protein dynamics and host-context-dependent functional performance. Because the tested *fos* genes originated from different staphylococcal species but were expressed in a common *S. aureus* background, native host-specific expression or biochemical context may also influence their apparent activity, as reported for other resistance enzymes such as OXA-type β-lactamases^26^. These possibilities remain mechanistic hypotheses and will require validation through residue-level mutagenesis, purified-enzyme kinetics and native-host experiments in the future.

Several limitations should be acknowledged. First, this study relied on publicly available genomes, whose representation varied across *Staphylococcus* species. Although genome-quality filtering and subsampling supported the robustness of the major distribution patterns, prevalence estimates and rare *fos* lineages should be interpreted in the context of current genome availability. Second, draft assemblies may limit complete reconstruction of plasmids, prophages and other mobile elements; therefore, prophage mobility was inferred from genomic context and requires experimental validation. Third, functional assays were performed in a heterologous *S. aureus* background, and structural modelling remains predictive; native-host validation, mutagenesis and enzyme-kinetic assays will be needed to define the underlying mechanisms. Future studies should also examine whether representative intrinsic *fos* genes affect bacterial physiology under non-antibiotic conditions, including oxidative or electrophilic stress, host-derived antimicrobial factors and ecological competition.

In conclusion, this study establishes a systematic nomenclature and classification framework for the *fos* gene family in staphylococci and reveals that *fos* determinants are distributed across both lineage-associated chromosomal backgrounds and multiple mobile reservoirs, including prophage-associated elements. These findings highlight the need to annotate both intrinsic and mobile *fos* determinants accurately when assessing fosfomycin resistance risk in clinically relevant staphylococci. Beyond improving annotation and surveillance, our findings raise the possibility that some *fos* genes are maintained for broader physiological functions than fosfomycin detoxification alone, providing a basis for future studies on their roles in staphylococcal adaptation and fitness.

## Methods

### Selection of species and identification of *fos* homologues

The following clinically common *Staphylococcus* species were included in the bioinformatic analysis: *S. aureus, S. epidermidis, S. argenteus, S. capitis, S. saprophyticus, S. warneri, S. lugdunensis, S. cohnii, S. caprae, S. haemolyticus, S. hominis, S. chromogenes, S. simulans and S. carnosus*. All genomes (18,839) from these 14 species that were available in the NCBI RefSeq Genome database (https://www.ncbi.nlm.nih.gov/genome) up to July 2023 were downloaded for further analysis. Assemblies were retained if they met the following criteria: CheckM completeness ≥95%, contamination ≤3%, assembly contig number <200 and contig N50 > 50 kb. Species-specific adjustment was applied for *S. hominis* because of its assembly characteristics. Genome-quality metrics and assembly statistics are provided in Supplementary Data 7.

Genomes of the selected *Staphylococcus* species were screened for *fos* gene homologues using *fosB*, *fosD*, *fosY* and *fosSA* as reference sequences reported in previous studies. These reference nucleotide sequences were applied to search across 17,024 *Staphylococcus* genomes available in the NCBI RefSeq database. BLASTN v2.9.0 was performed under thresholds of 40% nucleotide identity, 40% query coverage and a minimum aligned length of 210 bp. Candidate hits were extracted and translated into amino-acid sequences^12^. The translated sequences were then aligned using MUSCLE^27^ in Geneious Prime (https://www.geneious.com) and further evaluated by amino-acid identity, conserved motif inspection, phylogenetic placement and genomic-context analysis. A two-step strategy was employed: in the first round, candidate homologues were identified directly from the reference queries, and in the second round, sequences detected in the first iteration were used as additional queries to capture potential homologues overlooked initially^12^. Accession numbers of the reference *fos* sequences used for homologue searches and the corresponding nomenclature cross-reference are provided in Supplementary Data 1.

### Classification and definition of *fos* genes

While previous studies have identified multiple *fos* gene variants in staphylococci, the criteria for defining and naming these genes have often been inconsistent, complicating comparisons across datasets. To address this issue, we established a hierarchical classification framework combining sequence similarity thresholds and phylogenetic inference. Following published guidelines^28–30^, alleles were defined as sequences sharing ≥95% amino acid identity, gene families as groups sharing 90–95% identity, and identities <90% as distinct *fos* gene types. For groups close to these thresholds, particularly prophage-associated variants, maximum-likelihood phylogenies and pairwise amino-acid identity comparisons were used to refine lineage assignment. Borderline groups were classified according to concordance among identity thresholds, within- versus between-group identity patterns and phylogenetic topology, with bootstrap support ≥70% considered supportive of a phylogenetic clade. Detailed procedures for rare-candidate validation and threshold-adjacent classification are provided in the Supplementary Methods.

In addition to sequence-based classification, *fos* genes were further divided into intrinsic or acquired categories according to a combined genomic framework based on prevalence within species or major lineages, distribution pattern, local genomic context and association with mobile genetic elements. Genes with high prevalence within a species or lineage and conserved local genomic context were classified as intrinsic, whereas genes with sporadic distribution, cross-lineage or cross-species occurrence and variable mobile-element-associated contexts were classified as acquired.

### Assessment of sampling bias by species-level subsampling

To evaluate whether uneven species representation in public genome databases influenced the observed *fos* distribution patterns, we performed species-level subsampling after genome quality control, *fos* homologue identification and gene classification. For each species, up to 120 genomes were randomly selected, whereas all genomes were retained for species with fewer than 120 available genomes. This procedure was repeated ten times. The distribution of intrinsic and acquired *fos* genes in each subsampled dataset was compared with that in the full QC-filtered dataset.

### Phylogenetic analysis

All identified *fos* homologues, together with the reference sequences, were included in the phylogenetic reconstruction. Amino acid sequences were aligned using MUSCLE^27^ implemented in Geneious Prime (https://www.geneious.com), and the resulting alignment file was used to infer phylogeny with RAxML v8.2.11^31^. The maximum-likelihood method was applied under the LG + GAMMA substitution model, with 1000 bootstrap replicates to assess branch support. The phylogenetic trees were subsequently visualised and annotated using the Interactive Tree of Life (iTOL) platform (https://itol.embl.de/).

For species-level phylogenetic reconstruction, genome assemblies of the corresponding *Staphylococcus* species were annotated using Prokka v1.14.6^32^. Core-gene alignments were generated using Panaroo v2.2.3^33^ and the maximum-likelihood phylogenetic tree was then inferred in IQ-TREE v2.0.3^34^, with the best-fit substitution model automatically determined and branch support evaluated using 1000 bootstrap replicates. The tree was midpoint-rooted and visualised with iTOL (https://itol.embl.de/).

### Comparative genomic-context analysis

Comparative analysis of genomic regions surrounding representative *fos* genes was performed using selected *fos*-positive and *fos*-negative genomes. Genomic regions containing *fos* loci and their flanking sequences were extracted and compared to assess local synteny, conserved chromosomal integration, prophage-associated insertion sites and the presence of mobility-related genes. Pairwise sequence similarities between homologous regions were visualised using Easyfig v2.2.3^35^. Genome or contig accessions used for genomic-context comparisons are listed in Supplementary Data 4.

### Cloning of *fos* genes

Based on the classification framework described above, all identified intrinsic *fos* genes were selected for cloning, together with a subset of prophage-associated variants. Previously reported *fosD* and *fosY* genes from our laboratory were included as controls. The intrinsic *fos* genes of *S. aureus* and *S. epidermidis* were amplified directly from laboratory-preserved strains, while the remaining *fos* genes were synthesised commercially (Sangon Biotech, Shanghai, China). Gene fragments were cloned into the shuttle vector pTSSCm-pcap^36^ using homologous recombination, and the resulting recombinant plasmids were first propagated in *E. coli* DH5α. Plasmid DNA was subsequently extracted and electroporated into *S. aureus* RN4220. Transformants were selected on tryptic soy agar plates supplemented with tetracycline (12.5 µg/ml), and successful constructs were verified by Sanger sequencing. The primers used for amplification and verification are listed in Supplementary Data 5. All bacterial strains used as PCR templates, commercially synthesised gene sources and recombinant plasmids used in this study are listed in Supplementary Data 6.

### Construction of *fosSA* deletion mutant

Deletion of the *fosSA* gene in *S. aureus* strain LAC was performed by allelic replacement using plasmid pKOR1^37^. Approximately 1-kbp DNA fragments upstream and downstream of *fosSA* were amplified and assembled into pKOR1 by multi-fragment homologous recombination. The recombinant plasmid was first introduced into *S. aureus* RN4220 by electroporation and subsequently transferred into strain LAC via bacteriophage transduction using phage 80α. Allelic replacement was achieved by homologous recombination, and candidate *ΔfosSA* mutants were verified by PCR amplification of regions flanking the *fosSA* locus and by confirming the loss of the pKOR1 plasmid. The deletion allele was further confirmed by Sanger sequencing, and the primers used for amplification and verification are listed in Supplementary Data 5. The bacteriophage used for transduction is listed in Supplementary Data 6.

### Antimicrobial susceptibility testing

Fosfomycin MIC testing was performed in triplicate using the agar dilution method^38^, and median fosfomycin MIC values are reported. Briefly, Mueller–Hinton agar plates were supplemented with glucose-6-phosphate at 25 mg/L, and fosfomycin was incorporated into the agar at two-fold serial concentrations. Bacterial suspensions were prepared from overnight cultures and adjusted to the required inoculum density according to CLSI recommendations before being spotted onto agar plates. Plates were incubated at 35 ± 2 °C for 18–24 h, and MICs were read as the lowest fosfomycin concentration that inhibited visible growth. Susceptibility to non-fosfomycin antibiotics was determined using the broth microdilution method according to CLSI recommendations^38^. Fosfomycin MICs were interpreted according to EUCAST criteria^39^, whereas other antibiotics were interpreted in accordance with CLSI breakpoints^38^. *S. aureus* ATCC 29213 was used as the quality-control strain.

### Growth-curve and time-kill assays

Growth-curve assays were performed using *S. aureus* RN4220 carrying the empty vector or representative *fos* expression constructs under fosfomycin exposure at 16 mg/L. Overnight cultures were diluted into fresh medium containing fosfomycin, and bacterial growth was monitored by measuring OD_600_ over 24 h. OD_600_ values were recorded at 10 min intervals, and values at 2 h intervals were used for graphical presentation.

For time-kill assays, *S. aureus* RN4220 carrying the empty vector or representative fos expression constructs was exposed to fosfomycin at 16 mg/L. The RN4220/pT culture without fosfomycin was included as the untreated growth control, whereas RN4220/pT with fosfomycin served as the fosfomycin-exposed empty-vector control. Samples were collected at the indicated time points, serially diluted, plated and enumerated as CFU/mL. Viable counts are presented as log10 CFU/mL.

Both assays were performed using *n* = 2 independent biological replicates, each started from an independent bacterial culture. Line graphs were generated using GraphPad Prism 10.5 to show bacterial growth and survival kinetics over time.

### Structural modelling and docking analysis

Protein structures of representative *fos* variants were predicted using the AlphaFold Server based on AlphaFold 3^40^. For each variant, the top-ranked model was selected for downstream analysis. Fosfomycin docking was performed using the CB-Dock2 web server^41^, which conducts blind docking by automatically identifying potential binding cavities and applying AutoDock Vina scoring. No constraints were applied during docking. For each protein, the top-ranked binding pose based on Vina score was selected. Predicted binding pockets and interacting residues were extracted and compared across variants to assess similarities and differences in substrate-binding configurations. Docking results were analysed qualitatively, and binding energies were used for relative comparison only.

### Statistics and reproducibility

No statistical method was used to predetermine sample size. Genome datasets were defined by the availability of RefSeq assemblies that met the inclusion and quality-control criteria described above. No data were excluded from the analyses after application of the predefined genome quality-control and sequence-screening criteria. The experiments were not randomised. The investigators were not blinded to allocation during experiments and outcome assessment. No inferential statistical tests were performed. Fosfomycin MIC testing was performed in triplicate, and median values are reported. Growth-curve and time-kill assays were performed using two independent biological replicates, each initiated from an independent bacterial culture.

### Reporting summary

Further information on research design is available in the Nature Portfolio Reporting Summary linked to this article.

## Supplementary information

Supplementary Information

Description of Additional Supplementary Files

Supplementary Data 1–6

Supplementary Data 7

Supplementary Data 8

Reporting Summary

Transparent Peer Review file

## Source data

Source Data

## Data Availability

All publicly available genome assemblies analysed in this study were downloaded from the NCBI RefSeq database. The accession numbers of these genomes, together with genome quality-control information and *fos* gene carriage results, are provided in Supplementary Data 7. The *fos*-family sequence catalogue generated in this study, including sequence metadata and the corresponding nucleotide and amino acid sequences in FASTA format, is provided in Supplementary Data 8. Accession numbers for the representative sequences used to compare the genomic contexts of *fos* genes are provided in Supplementary Data 4. Source data for the main figures are provided with this paper. Source data are provided with this paper.
