# Supplementary material for: Decoding the genomic repertoire of fosfomycin resistance genes in staphylococci
Source: Nat Commun. 2026 Sep 10;17:10706. doi: 10.1038/s41467-026-77511-2 (PMC13658217; doi:10.1038/s41467-026-77511-2)
Supplement: Supplementary file 1 [file 41467_2026_77511_MOESM1_ESM.pdf]

Supplementary Methods

Manual validation of candidate *fos* homologues

Candidate *fos* homologues identified by the homology-search strategy described in the Methods were manually curated before downstream classification. For rare or newly detected *fos* candidates, particularly those located in prophage-associated regions, the corresponding contigs and flanking regions were manually inspected to evaluate locus authenticity. Candidates were retained only when the surrounding genomic context supported their assignment as authentic *Staphylococcus* loci or prophage-associated regions. Specifically, flanking regions were examined for conserved staphylococcal genes consistent with the host genome and/or recognizable prophage-related modules, including integrase/recombinase, terminase, capsid, tail, primase or replication-associated genes. Candidates located on short contigs lacking informative flanking regions, or those with genomic contexts suggestive of assembly artefacts or contamination, were excluded from downstream analyses.

Curation of borderline *fos* lineages

For *fos* groups with amino-acid identities close to the nomenclature thresholds used in this study, final lineage assignment was refined using phylogenetic support in addition to pairwise identity. Amino-acid identity thresholds were used as practical guides, with approximately 90% identity guiding family-level assignment and approximately 95% identity guiding allele-level assignment within a *fos* family.

For groups close to either the family-level or allele-level thresholds, phylogenetic topology, bootstrap support and within- versus between-group identity

patterns were used to refine lineage assignment. Sequences with identities close to the family-level threshold were assigned to the same *fos* family when they clustered together with adequate phylogenetic support and showed higher within-group than between-group identity. Sequences near this threshold that formed separately supported branches were retained as separate *fos* lineages.

Within an established *fos* family, related sequences close to the allele-level threshold were assigned as allele-level variants when they shared approximately ≥95% amino-acid identity and formed a supported within-family cluster. Sequences below the allele-level threshold were retained as distinct within-family variants or closely related family-level lineages according to their phylogenetic placement and identity structure.

Assignment of intrinsic and acquired *fos* genes

After sequence-based classification, *fos* genes were categorized as intrinsic or acquired according to their distribution patterns, genomic contexts and inferred evolutionary behavior. Intrinsic *fos* genes were defined as determinants that were highly prevalent within a species or major lineage, embedded in conserved chromosomal architectures and lacked evidence of recent horizontal transfer. Genomic location alone was not used to define intrinsic status.

Acquired *fos* genes were defined by sporadic or cross-lineage distribution, occurrence across species boundaries, association with plasmids, genomic islands or prophages, variable flanking regions or insertion sites, and/or the presence of mobility-related modules. Prophage association alone was not considered sufficient to

classify a *fos* gene as acquired; acquired status was assigned only when prophage association was accompanied by distributional or genomic-context evidence consistent with horizontal transfer.

This intrinsic/acquired categorization also informed the reporting resolution of internal sequence diversity. For species- or lineage-associated intrinsic *fos* genes, internal diversity was generally retained as variant-level information when the genes showed conserved chromosomal context and stable host-associated distribution, in order to avoid over-fragmentation of intrinsic chromosomal determinants. For mobile-element-associated *fos* genes, especially prophage-associated determinants, closely related lineages were resolved more explicitly when pairwise identity and phylogenetic support supported family- or allele-level subdivision, because such subdivision was relevant to tracking mobile reservoirs and potential dissemination.

a

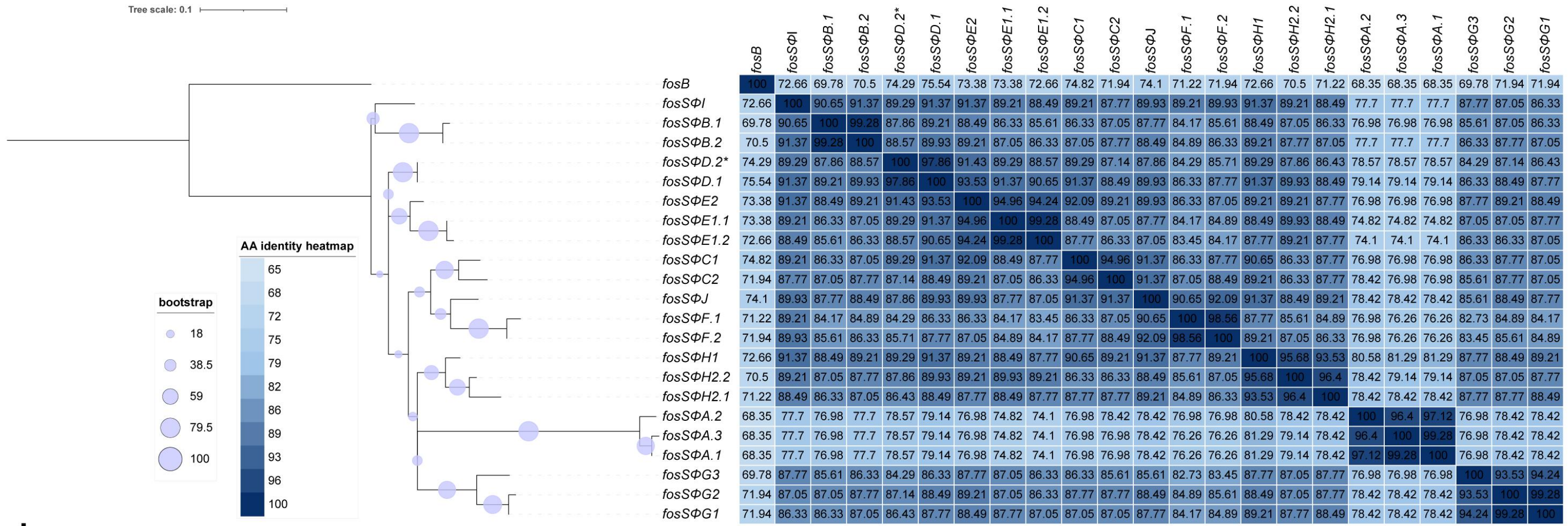

Tree scale: 0.1
AA identity heatmap
65
68
72
75
79
82
86
89
93
96
100
bootstrap
18
38.5
59
79.5
100
fosB fosSΦI fosSΦB.1 fosSΦB.2 fosSΦD.2* fosSΦD.1 fosSΦE2 fosSΦE1.1 fosSΦE1.2 fosSΦC1 fosSΦC2 fosSΦJ fosSΦF.1 fosSΦF.2 fosSΦH1 fosSΦH2.2 fosSΦH2.1 fosSΦA.2 fosSΦA.3 fosSΦA.1 fosSΦG3 fosSΦG2 fosSΦG1
fosB 100 72.66 69.78 70.5 74.29 75.54 73.38 73.38 72.66 74.82 71.94 74.1 71.22 71.94 72.66 70.5 71.22 68.35 68.35 68.35 69.78 71.94 71.94
fosSΦI 72.66 100 90.65 91.37 89.29 91.37 91.37 89.21 88.49 89.21 87.77 89.93 89.21 89.93 91.37 89.21 88.49 77.7 77.7 77.7 87.77 87.05 86.33
fosSΦB.1 69.78 90.65 100 99.28 87.86 89.21 88.49 86.33 85.61 86.33 87.05 87.77 84.17 85.61 88.49 87.05 86.33 76.98 76.98 76.98 85.61 87.05 86.33
fosSΦB.2 70.5 91.37 99.28 100 88.57 89.93 89.21 87.05 86.33 87.05 87.77 88.49 84.89 86.33 89.21 87.77 87.05 77.7 77.7 77.7 86.33 87.77 87.05
fosSΦD.2* 74.29 89.29 87.86 88.57 100 97.86 91.43 89.29 88.57 89.29 87.14 87.86 84.29 85.71 89.29 87.86 86.43 78.57 78.57 78.57 84.29 87.14 86.43
fosSΦD.1 75.54 91.37 89.21 89.93 97.86 100 93.53 91.37 90.65 91.37 88.49 89.93 86.33 87.77 91.37 89.93 88.49 79.14 79.14 79.14 86.33 88.49 87.77
fosSΦE2 73.38 91.37 88.49 89.21 91.43 93.53 100 94.96 94.24 92.09 89.21 89.93 86.33 87.05 89.21 89.21 87.77 76.98 76.98 76.98 87.77 89.21 88.49
fosSΦE1.1 73.38 89.21 86.33 87.05 89.29 91.37 94.96 100 99.28 88.49 87.05 87.77 84.17 84.89 88.49 89.93 88.49 74.82 74.82 74.82 87.05 87.05 87.77
fosSΦE1.2 72.66 88.49 85.61 86.33 88.57 90.65 94.24 99.28 100 87.77 86.33 87.05 83.45 84.17 87.77 89.21 87.77 74.1 74.1 74.1 86.33 86.33 87.05
fosSΦC1 74.82 89.21 86.33 87.05 89.29 91.37 92.09 88.49 87.77 100 94.96 91.37 86.33 87.77 90.65 86.33 87.77 76.98 76.98 76.98 86.33 87.77 87.05
fosSΦC2 71.94 87.77 87.05 87.77 87.14 88.49 89.21 87.05 86.33 94.96 100 91.37 87.05 88.49 89.21 86.33 87.77 78.42 76.98 76.98 85.61 87.77 87.05
fosSΦJ 74.1 89.93 87.77 88.49 87.86 89.93 89.93 87.77 87.05 91.37 91.37 100 90.65 92.09 91.37 88.49 89.21 78.42 78.42 78.42 85.61 88.49 87.77
fosSΦF.1 71.22 89.21 84.17 84.89 84.29 86.33 86.33 84.17 83.45 86.33 87.05 90.65 100 98.56 87.77 85.61 84.89 76.98 76.26 76.26 82.73 84.89 84.17
fosSΦF.2 71.94 89.93 85.61 86.33 85.71 87.77 87.05 84.89 84.17 87.77 88.49 92.09 98.56 100 89.21 87.05 86.33 76.98 76.26 76.26 83.45 85.61 84.89
fosSΦH1 72.66 91.37 88.49 89.21 89.29 91.37 89.21 88.49 87.77 90.65 89.21 91.37 87.77 89.21 100 95.68 93.53 80.58 81.29 81.29 87.77 88.49 89.21
fosSΦH2.2 70.5 89.21 87.05 87.77 87.86 89.93 89.21 89.93 89.21 86.33 86.33 88.49 85.61 87.05 95.68 100 96.4 78.42 79.14 79.14 87.05 87.05 87.77
fosSΦH2.1 71.22 88.49 86.33 87.05 86.43 88.49 87.77 88.49 87.77 87.77 87.77 89.21 84.89 86.33 93.53 96.4 100 78.42 78.42 78.42 87.77 87.77 88.49
fosSΦA.2 68.35 77.7 76.98 77.7 78.57 79.14 76.98 74.82 74.1 76.98 78.42 78.42 76.98 76.98 80.58 78.42 78.42 100 96.4 97.12 76.98 78.42 78.42
fosSΦA.3 68.35 77.7 76.98 77.7 78.57 79.14 76.98 74.82 74.1 76.98 76.98 78.42 76.26 76.26 81.29 79.14 78.42 96.4 100 99.28 76.98 78.42 78.42
fosSΦA.1 68.35 77.7 76.98 77.7 78.57 79.14 76.98 74.82 74.1 76.98 76.98 78.42 76.26 76.26 81.29 79.14 78.42 97.12 99.28 100 76.98 78.42 78.42
fosSΦG3 69.78 87.77 85.61 86.33 84.29 86.33 87.77 87.05 86.33 86.33 85.61 85.61 82.73 83.45 87.77 87.05 87.77 76.98 76.98 76.98 100 93.53 94.24
fosSΦG2 71.94 87.05 87.05 87.77 87.14 88.49 89.21 87.05 86.33 87.77 87.77 88.49 84.89 85.61 88.49 87.05 87.77 78.42 78.42 78.42 93.53 100 99.28
fosSΦG1 71.94 86.33 86.33 87.05 86.43 87.77 88.49 87.77 87.05 87.05 87.05 87.77 84.17 84.89 89.21 87.77 88.49 78.42 78.42 78.42 94.24 99.28 100

b

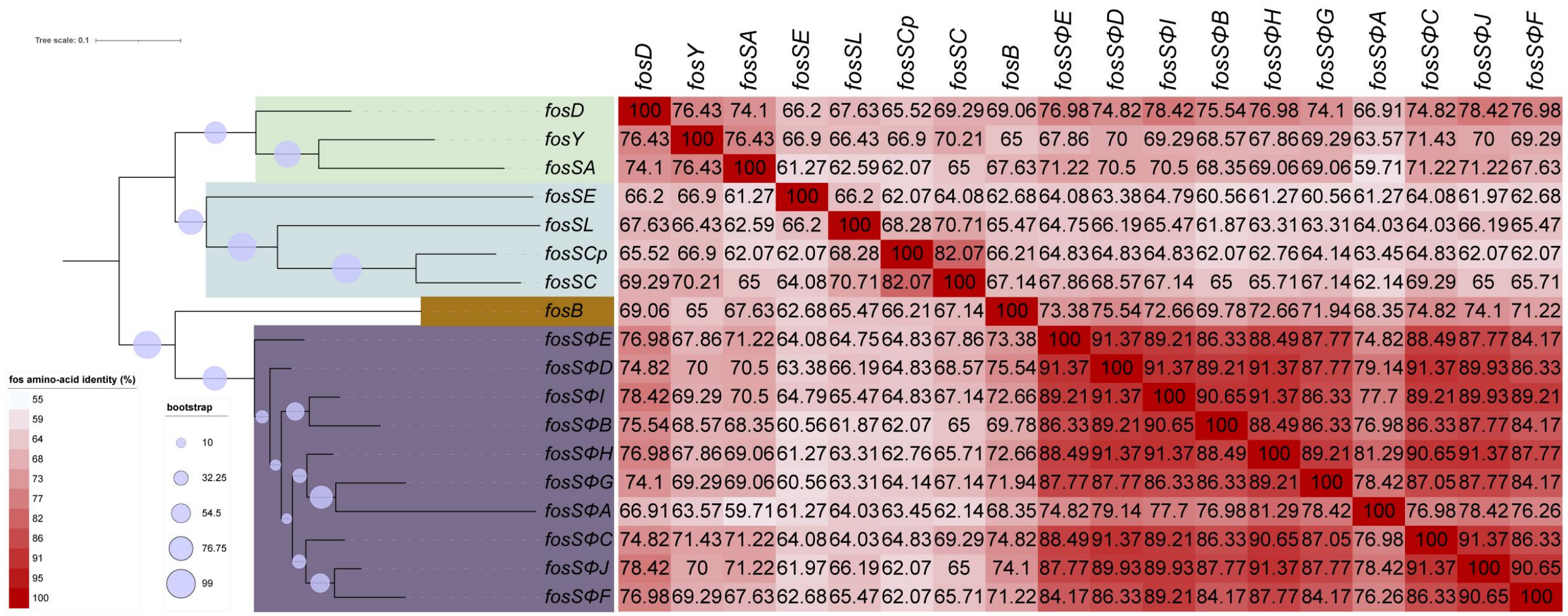

Tree scale: 0.1
fos amino-acid identity (%)
55
59
64
68
73
77
82
86
91
95
100
bootstrap
10
32.25
54.5
76.75
99
fosD fosY fosSA fosSE fosSL fosSCp fosSC fosB fosSΦE fosSΦD fosSΦI fosSΦB fosSΦH fosSΦG fosSΦA fosSΦC fosSΦJ fosSΦF
fosD 100 76.43 74.1 66.2 67.63 65.52 69.29 69.06 76.98 74.82 78.42 75.54 76.98 74.1 66.91 74.82 78.42 76.98
fosY 76.43 100 76.43 66.9 66.43 66.9 70.21 65 67.86 70 69.29 68.57 67.86 69.29 63.57 71.43 70 69.29
fosSA 74.1 76.43 100 61.27 62.59 62.07 65 67.63 71.22 70.5 70.5 68.35 69.06 69.06 59.71 71.22 71.22 67.63
fosSE 66.2 66.9 61.27 100 66.2 62.07 64.08 62.68 64.08 63.38 64.79 60.56 61.27 60.56 61.27 64.08 61.97 62.68
fosSL 67.63 66.43 62.59 66.2 100 68.28 70.71 65.47 64.75 66.19 65.47 61.87 63.31 63.31 64.03 64.03 66.19 65.47
fosSCp 65.52 66.9 62.07 62.07 68.28 100 82.07 66.21 64.83 64.83 64.83 62.07 62.76 64.14 63.45 64.83 62.07 62.07
fosSC 69.29 70.21 65 64.08 70.71 82.07 100 67.14 67.86 68.57 67.14 65 65.71 67.14 62.14 69.29 65 65.71
fosB 69.06 65 67.63 62.68 65.47 66.21 67.14 100 73.38 75.54 72.66 69.78 72.66 71.94 68.35 74.82 74.1 71.22
fosSΦE 76.98 67.86 71.22 64.08 64.75 64.83 67.86 73.38 100 91.37 89.21 86.33 88.49 87.77 74.82 88.49 87.77 84.17
fosSΦD 74.82 70 70.5 63.38 66.19 64.83 68.57 75.54 91.37 100 91.37 89.21 91.37 87.77 79.14 91.37 89.93 86.33
fosSΦI 78.42 69.29 70.5 64.79 65.47 64.83 67.14 72.66 89.21 91.37 100 90.65 91.37 86.33 77.7 89.21 89.93 89.21
fosSΦB 75.54 68.57 68.35 60.56 61.87 62.07 65 69.78 86.33 89.21 90.65 100 88.49 86.33 76.98 86.33 87.77 84.17
fosSΦH 76.98 67.86 69.06 61.27 63.31 62.76 65.71 72.66 88.49 91.37 91.37 88.49 100 89.21 81.29 90.65 91.37 87.77
fosSΦG 74.1 69.29 69.06 60.56 63.31 64.14 67.14 71.94 87.77 87.77 86.33 86.33 89.21 100 78.42 87.05 87.77 84.17
fosSΦA 66.91 63.57 59.71 61.27 64.03 63.45 62.14 68.35 74.82 79.14 77.7 76.98 81.29 78.42 100 76.98 78.42 76.26
fosSΦC 74.82 71.43 71.22 64.08 64.03 64.83 69.29 74.82 88.49 91.37 89.21 86.33 90.65 87.05 76.98 100 91.37 86.33
fosSΦJ 78.42 70 71.22 61.97 66.19 62.07 65 74.1 87.77 89.93 89.93 87.77 91.37 87.77 78.42 91.37 100 90.65
fosSΦF 76.98 69.29 67.63 62.68 65.47 62.07 65.71 71.22 84.17 86.33 89.21 84.17 87.77 84.17 76.26 86.33 90.65 100

Supplementary Figure 1 Phylogenetic relationships and pairwise amino-acid identity patterns of representative *fos* genes.

Phylogenetic trees and pairwise amino-acid identity heatmaps of representative prophage-associated *fosSΦ* genes (**a**) and major *fos* families (**b**). Trees were reconstructed from aligned Fos amino-acid sequences, and bootstrap support is indicated by circle size at internal nodes. Pairwise amino-acid identity values (%) were calculated using Geneious Prime. In panel a, asterisks indicate representative genes containing premature stop codons.

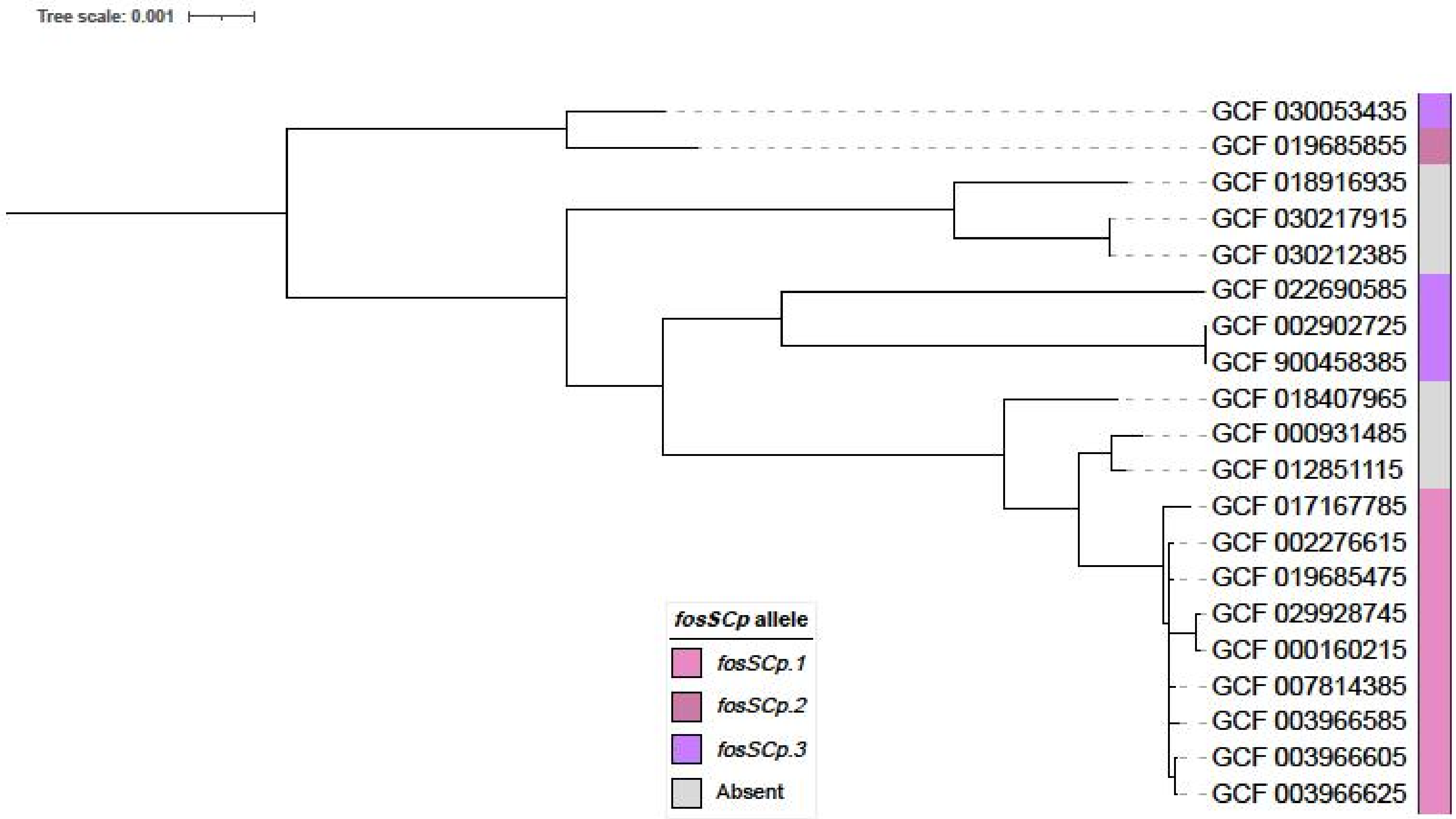

Supplementary Figure 2 Clade-associated distribution of *fosSCp* in *Staphylococcus caprae*.

Core-genome phylogeny of *S. caprae* genomes showing the distribution of *fosSCp*. Coloured strips indicate *fosSCp* alleles, whereas grey indicates absence of detectable *fosSCp*.

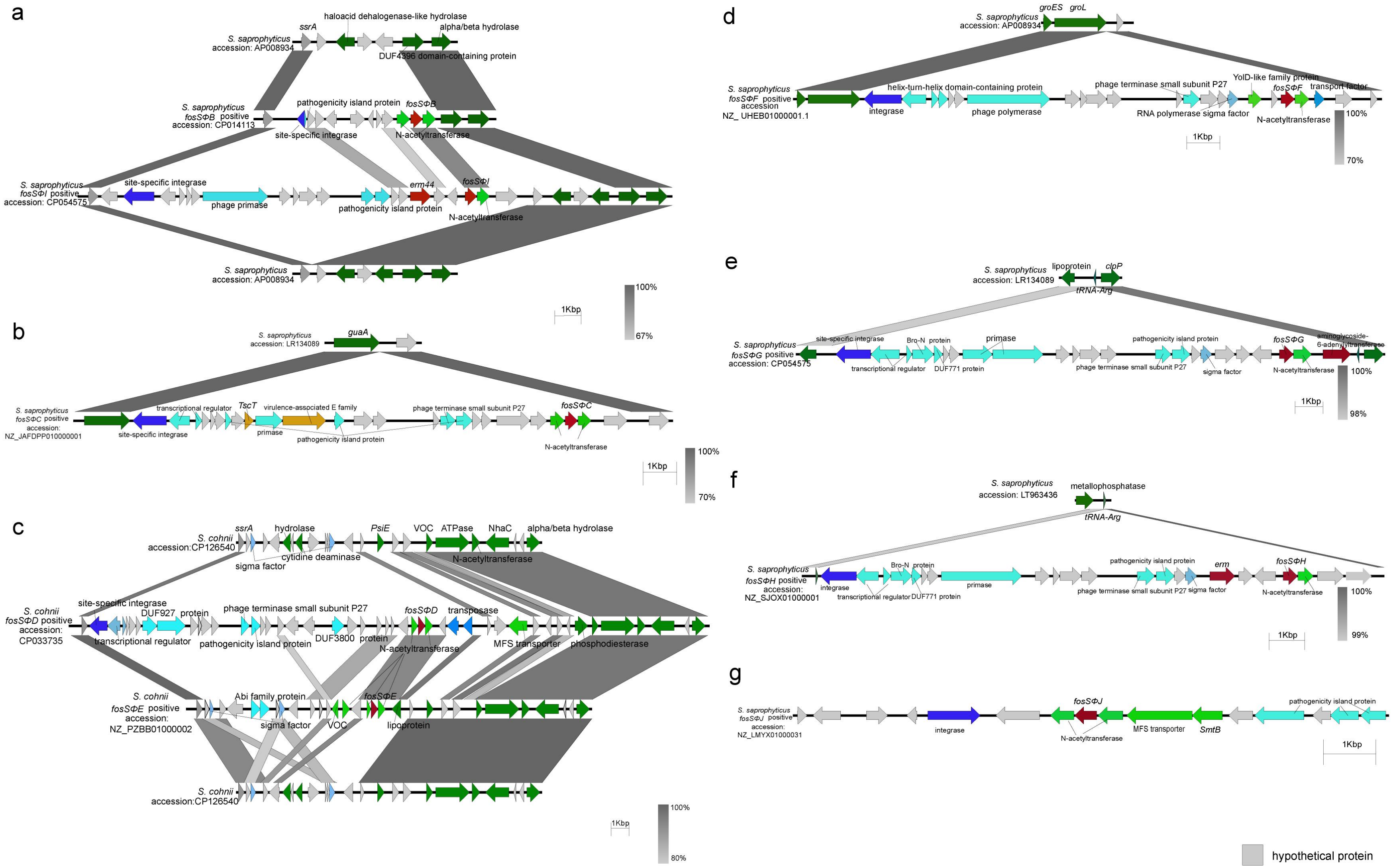
a
S. saprophyticus accession: AP008934
ssrA
haloacid dehalogenase-like hydrolase
alpha/beta hydrolase
DUF4396 domain-containing protein
S. saprophyticus fosSΦB positive accession: CP014113
pathogenicity island protein
fosSΦB
site-specific integrase
N-acetyltransferase
S. saprophyticus fosSΦI positive accession: CP054575
site-specific integrase
phage primase
erm44
fosSΦI
pathogenicity island protein
N-acetyltransferase
S. saprophyticus accession: AP008934
100%
67%
1Kbp
b
S. saprophyticus accession: LR134089
guaA
S. saprophyticus fosSΦC positive accession: NZ_JAFDPP010000001
transcriptional regulator
TscT
virulence-associated E family
phage terminase small subunit P27
fosSΦC
site-specific integrase
primase
pathogenicity island protein
N-acetyltransferase
100%
70%
1Kbp
c
S. cohnii accession:CP126540
ssrA
hydrolase
PsiE
VOC
ATPase
NhaC
alpha/beta hydrolase
sigma factor
cytidine deaminase
N-acetyltransferase
S. cohnii fosSΦD positive accession: CP033735
site-specific integrase
DUF927 protein
phage terminase small subunit P27
fosSΦD
transposase
transcriptional regulator
pathogenicity island protein
DUF3800 protein
N-acetyltransferase
MFS transporter
phosphodiesterase
S. cohnii fosSΦE positive accession: NZ_PZBB01000002
Abi family protein
fosSΦE
sigma factor
VOC
lipoprotein
S. cohnii accession:CP126540
100%
80%
1Kbp
d
S. saprophyticus accession: AP008934
groES
groL
S. saprophyticus fosSΦF positive accession NZ_UHEB01000001.1
helix-turn-helix domain-containing protein
phage terminase small subunit P27
YolD-like family protein
fosSΦF
transport factor
integrase
phage polymerase
RNA polymerase sigma factor
N-acetyltransferase
100%
70%
1Kbp
e
S. saprophyticus accession: LR134089
lipoprotein
clpP
tRNA-Arg
S. saprophyticus fosSΦG positive accession: CP054575
site-specific integrase
Bro-N protein
primase
pathogenicity island protein
fosSΦG
aminoglycoside-6-adenyltransferase
transcriptional regulator
DUF771 protein
phage terminase small subunit P27
sigma factor
N-acetyltransferase
100%
98%
1Kbp
f
S. saprophyticus accession: LT963436
metallophosphatase
tRNA-Arg
S. saprophyticus fosSΦH positive accession: NZ_SJOX01000001
integrase
Bro-N protein
primase
pathogenicity island protein
erm
fosSΦH
transcriptional regulator
DUF771 protein
phage terminase small subunit P27
sigma factor
N-acetyltransferase
100%
99%
1Kbp
g
S. saprophyticus fosSΦJ positive accession: NZ_LMYX01000031
integrase
fosSΦJ
N-acetyltransferase
MFS transporter
SmtB
pathogenicity island protein
1Kbp
hypothetical protein

Supplementary Figure 3. Genomic contexts of prophage-associated *fosSΦ* genes.

a, Genomic contexts of *fosSΦB* and *fosSΦI* in *Staphylococcus saprophyticus*. **b,** Genomic context of *fosSΦC* in *S. saprophyticus*. **c,** Genomic contexts of *fosSΦD* and *fosSΦE* in *Staphylococcus cohnii*. **d,** Genomic context of *fosSΦF* in *S. saprophyticus*. **e,** Genomic context of *fosSΦG* in *S. saprophyticus*. **f,** Genomic context of *fosSΦH* in *S. saprophyticus*. **g,** Genomic context of *fosSΦJ* in *S. saprophyticus*. For all panels, genes with different functional categories are indicated by different colours, and the corresponding *fosSΦ* genes are highlighted in red. Grey shading denotes nucleotide sequence similarity between homologous regions. Accession numbers of the genomes or contigs used for comparison are shown in each panel. Scale bars, 1 kb.
