# Supplementary material for: Decoding the genomic repertoire of fosfomycin resistance genes in staphylococci
Source: Nat Commun. 2026 Sep 10;17:10706. doi: 10.1038/s41467-026-77511-2 (PMC13658217; doi:10.1038/s41467-026-77511-2)
Supplement: Supplementary file 2 [file 41467_2026_77511_MOESM2_ESM.pdf]

Description of Additional Supplementary Files

File Name: Supplementary Data 1
Description: Reference sequences used for *fos* homologue searches and a cross-reference between previous annotations and the nomenclature proposed in this study, including representative accessions, host species, gene category, typical genomic context, initial BLAST-query status and the rationale for nomenclature.

File Name: Supplementary Data 2
Description: Species-level subsampling analysis used to assess the effect of uneven genome representation on *fos* gene distribution, including full-dataset prevalence and summary statistics from ten random subsampling replicates.

File Name: Supplementary Data 3
Description: Fosfomycin minimum inhibitory concentrations, predicted AutoDock Vina binding energies, pocket classifications and key predicted substrate-accommodation residues for representative Fos variants.

File Name: Supplementary Data 4
Description: Genome or contig accessions used for comparative genomic-context analyses, together with strain descriptions, *fos*-associated regions and *fos*-positive or *fos*-negative status.

File Name: Supplementary Data 5
Description: Oligonucleotide primer names, 5’–3’ sequences and applications used for *fosSA* deletion, *fos* gene cloning and construct verification.

File Name: Supplementary Data 6
Description: Bacterial strains, bacteriophage, plasmids and synthetic gene sources used in this study, including their genetic background, relevant features, experimental purpose and source or reference.

File Name: Supplementary Data 7
Description: Genome-level metadata, assembly accessions, genome-quality metrics and *fos* gene carriage or allele status for 17,024 quality-filtered genomes representing 14 *Staphylococcus* species. The workbook contains a README worksheet and separate species-specific worksheets.

File Name: Supplementary Data 8
Description: ZIP archive containing an Excel file with metadata for 151 curated *fos*-family sequences, including gene and family names, host species, accession numbers, coding-sequence coordinates, strand orientation and sequence length, together with two FASTA files containing the corresponding nucleotide and amino acid sequences.
