# Supplementary material for: Decoding the genomic repertoire of fosfomycin resistance genes in staphylococci
Source: Nat Commun. 2026 Sep 10;17:10706. doi: 10.1038/s41467-026-77511-2 (PMC13658217; doi:10.1038/s41467-026-77511-2)
Supplement: Supplementary file 3 [file 41467_2026_77511_MOESM6_ESM.pdf]

nature portfolio

Corresponding author(s): Yan Chen

Last updated by author(s): Jul 3, 2026

Reporting Summary

Nature Portfolio wishes to improve the reproducibility of the work that we publish. This form provides structure for consistency and transparency in reporting. For further information on Nature Portfolio policies, see our Editorial Policies and the Editorial Policy Checklist.

Statistics

For all statistical analyses, confirm that the following items are present in the figure legend, table legend, main text, or Methods section.

n/a	Confirmed	
☐	☒	The exact sample size (n) for each experimental group/condition, given as a discrete number and unit of measurement
☐	☒	A statement on whether measurements were taken from distinct samples or whether the same sample was measured repeatedly
☒	☐	The statistical test(s) used AND whether they are one- or two-sided<br>*Only common tests should be described solely by name; describe more complex techniques in the Methods section.*
☒	☐	A description of all covariates tested
☒	☐	A description of any assumptions or corrections, such as tests of normality and adjustment for multiple comparisons
☐	☒	A full description of the statistical parameters including central tendency (e.g. means) or other basic estimates (e.g. regression coefficient) AND variation (e.g. standard deviation) or associated estimates of uncertainty (e.g. confidence intervals)
☒	☐	For null hypothesis testing, the test statistic (e.g. F, t, r) with confidence intervals, effect sizes, degrees of freedom and P value noted<br>*Give P values as exact values whenever suitable.*
☒	☐	For Bayesian analysis, information on the choice of priors and Markov chain Monte Carlo settings
☒	☐	For hierarchical and complex designs, identification of the appropriate level for tests and full reporting of outcomes
☒	☐	Estimates of effect sizes (e.g. Cohen's d, Pearson's r), indicating how they were calculated

Our web collection on statistics for biologists contains articles on many of the points above.

Software and code

Policy information about availability of computer code

Data collection	Genome assemblies were retrieved from the NCBI RefSeq Genome database up to July 2023. No dedicated data-collection software was used beyond database retrieval from NCBI RefSeq.
Data analysis	fos homologues were identified using BLASTN v2.9.0. Amino-acid alignments were generated using MUSCLE in Geneious Prime, and fos-family phylogenies were inferred with RAxML v8.2.11 under the LG+GAMMA model with 1,000 bootstrap replicates. For species-level phylogenetic reconstruction, genome assemblies were annotated using Prokka v1.14.6, core-gene alignments were generated using Panaroo v2.2.3, and maximum-likelihood trees were inferred using IQ-TREE v2.0.3 with automatic model selection and 1,000 bootstrap replicates. Genomic-context comparisons were visualized using Easyfig v2.2.3. Tree visualization and annotation were performed using iTOL. Growth-curve and time-kill graphs were generated using GraphPad Prism 10.5. Protein structures were predicted using AlphaFold Server based on AlphaFold 3, and fosfomycin docking was performed using the CB-Dock2 web server.

For manuscripts utilizing custom algorithms or software that are central to the research but not yet described in published literature, software must be made available to editors and reviewers. We strongly encourage code deposition in a community repository (e.g. GitHub). See the Nature Portfolio guidelines for submitting code & software for further information.

Data

Policy information about availability of data

All manuscripts must include a data availability statement. This statement should provide the following information, where applicable:
- Accession codes, unique identifiers, or web links for publicly available datasets
- A description of any restrictions on data availability
- For clinical datasets or third party data, please ensure that the statement adheres to our policy

All publicly available genome assemblies analysed in this study were downloaded from the NCBI RefSeq database. The accession numbers of these genomes, together with genome quality-control information and fos gene carriage results, are provided in Supplementary Data 7. The fos-family sequence catalogue generated in this study, including sequence metadata and the corresponding fos amino-acid sequences in FASTA format, is provided in Supplementary Data 8. Accession numbers for the representative sequences used to compare the genomic contexts of fos genes are provided in Supplementary Data 4. Source data for the main figures are provided with this paper.

Research involving human participants, their data, or biological material

Policy information about studies with human participants or human data. See also policy information about sex, gender (identity/presentation), and sexual orientation and race, ethnicity and racism.

Reporting on sex and gender	not applicable
Reporting on race, ethnicity, or other socially relevant groupings	not applicable
Population characteristics	not applicable
Recruitment	not applicable
Ethics oversight	not applicable

Note that full information on the approval of the study protocol must also be provided in the manuscript.

Field-specific reporting

Please select the one below that is the best fit for your research. If you are not sure, read the appropriate sections before making your selection.

☒ Life sciences ☐ Behavioural & social sciences ☐ Ecological, evolutionary & environmental sciences

For a reference copy of the document with all sections, see nature.com/documents/nr-reporting-summary-flat.pdf

Life sciences study design

All studies must disclose on these points even when the disclosure is negative.

Sample size	No statistical method was used to predetermine sample size. Genome sample sizes were determined by the availability of NCBI RefSeq assemblies from the selected Staphylococcus species that met the predefined inclusion and quality-control criteria. Fosfomycin MIC testing was performed in triplicate, and growth-curve and time-kill assays were performed using two independent biological replicates.
Data exclusions	Assemblies were retained if they met the following criteria: CheckM completeness ≥95%, contamination ≤3%, assembly contig number < 200 and contig N50 > 50 kb.
Replication	Reproducibility was supported by predefined genome quality-control and sequence-screening criteria, repeated species-level subsampling, triplicate fosfomycin MIC testing, independent biological replicates for growth-curve and time-kill assays, and PCR/Sanger verification of genetic constructs.
Randomization	The experiments were not randomized. Genome inclusion was based on availability in NCBI RefSeq and predefined quality-control criteria. Random selection was used only for species-level subsampling to assess the effect of uneven species representation.
Blinding	The investigators were not blinded to allocation during experiments and outcome assessment. Most analyses were based on predefined computational criteria, and laboratory assays involved predefined strain and construct groups.

Reporting for specific materials, systems and methods

We require information from authors about some types of materials, experimental systems and methods used in many studies. Here, indicate whether each material, system or method listed is relevant to your study. If you are not sure if a list item applies to your research, read the appropriate section before selecting a response.

Materials & experimental systems

n/a	Involved in the study
☒	☐ Antibodies
☒	☐ Eukaryotic cell lines
☒	☐ Palaeontology and archaeology
☒	☐ Animals and other organisms
☒	☐ Clinical data
☒	☐ Dual use research of concern
☒	☐ Plants

Methods

n/a	Involved in the study
☒	☐ ChIP-seq
☒	☐ Flow cytometry
☒	☐ MRI-based neuroimaging

Plants

Seed stocks	Not applicable.
Novel plant genotypes	Not applicable.
Authentication	Not applicable.
