# Supplementary material for: Decoding the genomic repertoire of fosfomycin resistance genes in staphylococci
Source: Nat Commun. 2026 Sep 10;17:10706. doi: 10.1038/s41467-026-77511-2 (PMC13658217; doi:10.1038/s41467-026-77511-2)
Supplement: Supplementary file 4 [file 41467_2026_77511_MOESM7_ESM.pdf]

nature portfolio

Peer Review File

Decoding the genomic repertoire of fosfomycin resistance genes in staphylococci

Corresponding Author: Dr Yan Chen

This file contains all reviewer reports in order by version, followed by all author rebuttals in order by version.

Version 0:

Reviewer comments:

Reviewer #1

(Remarks to the Author)
The manuscript addresses fosfomycin resistance in Staphylococci, the genes involved and their nomenclature. It identifies novel genes and demonstrates that they increase fosfomycin resistance. In general, it would be useful with more information about the putative mode of action of the different resistance genes and if that was taken into account when forming the resistance gene nomenclature. Also did you observe any correlations between predicted modes of action and gene locations? Furthermore the authors do not address if there is any effect of Fosfomycin resistance and B-lactam resistance which may be predicted based on the mode of action of Fosfomycin.
Abstract: In general, the novelty and impact of the manuscript should be highlighted to a greater extent as there are other examples of resistance genes being present both in the core genome and present on various mobile genetic elements. Also the compound Fosfomycin should be introduced what it targets.
L 28: Change wording (the genes are not confused) and mention what exactly is confusing
L 30: were these species selected for a reason?
L 38: MIC is not defined
L 43: Silent fos genes – does this mean that the genes are not providing resistance? The goes against the statement in line 39 about a 16 fold reduction in MIC

Main text:
L 62: Expand on the description of resistance – what do you mean by defects?
L 68-78: Are these gene products providing resistance via glpT or uhpT?
L 188: Provide an argument for selecting these species?
L 214-218: Mention if the type of resistance correlates with location (core, mobile, phage)
L. 213: Did you pay attention to putative mode of action when assigning gene names?
L278-279: with the report here or prior?
L. 327: mention if they are cloned with their own promoter or common heterologous promoters
L. 335-341: Given that Fosfomycin targets the UDP-N-acetylglucosamine-3-enolpyruvyltransferase there could be an interplay with B-lactam susceptibility – this should be tested for all your constructs.

Reviewer #2

(Remarks to the Author)
Happy to have the opportunity to review the manuscript entitled "Decoding a silent reservoir of fosfomycin resistance genes in Staphylococcus: A genomic and evolutionary study".

Title. To begin, I do not quite understand the title. Why is it a "silent reservoir"? The genes seem to provide some low/moderate level resistance to fosfomycin and they have other physiological functions within the cell. Why silent?

Abstract. As above "this work uncovers a previously hidden ... reservoir of silent fos genes..." were they hidden because they only have a small impact of fosfomycin susceptibility? Please try to clarify.

Intro. The intro should give some introduction to the function of the enzyme coded by fos genes. What does it do?

Line 56 - I think these two references should be discussed with more care/caution. There was no statistically significant improvement compared to monotherapy when fosfomycin was included in those studies.
Line 68 - the sentence is not clear to me, "with amino acid identities ranging from 62% to 80%," compared to what?
Line 76 - "Notably, many researchers have failed to distinguish..." This warrants some type of reference/some evidence.
Small point - Staphylococci is not the genus name, so it is not in italics, Staphylococcus is the genus name and should be in italics. Also, fos, indicative of a gene, should be consistently in italics.

Materials methods. Why were only 14 species used? Without a rationale for exclusion I think more species should be included.
Provide refs for Snippy and iToL
Does RN4220 have a fos gene?
What media was used for the MIC - was it according to CLSI/EUCAST guidance?

Results - can you relate phylogeny of the intrinsic fos genes with general phylogeny of the species. I guess it would be similar?

Line 226-232 and Figure 2A - Can a comment be made on the frequency of those ST in, for example, hospital environments, lifestock, etc environments? Could a particular fos allele be associated with "successful/prominent lineages" of S. aureus?

To help interpret the figure, is the relative size of each band on the left of Figure 2A indicative of the relative proportion of each ST in your analysis? I think could be mentioned in the legend.

Figure 2 - It is odd that the same type of information across the 5 panels is presented in 5 different ways. Makes it difficult to compare across the species. Why are only 5 of the 14 species assessed in this type of analysis? At least a comment should be made as to why the others did not get the same assessment.

Line 273 - there is a word missing in this sentence (possibly "...almost ALL currently...")

Line 322 - what evidence do you have that the genes are cryptic? For example, fosSC is not a cryptic gene. When it is present, the MIC for fosfomycin is very high https://www.sciencedirect.com/science/article/pii/S0924857924000803. Not sure what is cryptic about that.

The fosfomycin MICs for Table 2 should be the median of multiple measurements (with range included, if appropriate). This can help to reduce the potential impact of intrinsic error associated with the agar dilution method.

Reviewer #3

(Remarks to the Author)
This manuscript presents a comprehensive genomic analysis of 18,839 Staphylococcus genomes, uncovering a previously unrecognized diversity of fos genes associated with fosfomycin resistance. The authors introduce a novel identification and classification framework for fos gene families and report five intrinsic chromosomal determinants alongside eight novel prophage-associated families, with variable levels of resistance through distinct evolutionary trajectories and horizontal gene transfer mechanisms that threaten the clinical utility of fosfomycin.
Overall, this is a useful and well-structured study. However, several important clarifications and methodological details are needed before the conclusions can be fully supported.

MAJOR comments:
The observed diversity of fos gene families appears highly dependent on the underlying sampling distribution of genomes. The authors apply a retention criterion of alleles present in ≥3 genomes; however, the sampling across Staphylococcus species is uneven. This could inflate signals from overrepresented species and lineages while filtering out rare determinants, leading to underestimation of novel gene families.
Please consider implementing normalization (e.g., subsampling or weighted inference) to mitigate sampling bias and clarify how uneven sampling may affect claims of diversity and ubiquity across species.
The methods section lacks details on the quality control of the 18,839 genomes obtained from NCBI. Were all genomes complete assemblies? Were plasmid sequences available for all isolates? Since assembly completeness and plasmid representation can directly influence the detection of fos loci, these details are essential. Please include metrics such as completeness, contamination, and assembly statistics in the supplementary materials.
It is critical to ensure that the 164 identified fos sequences originate from Staphylococcus assemblies and not from contaminants or misassembled contigs. Please describe any QC steps or filtering criteria used to verify the authenticity of these sequences.
Because of the highly uneven number of genomes analyzed across species, it is important to consistently include the total number of genomes (n) whenever percentages are reported throughout the Results section.
The categorization of intrinsic v/s acquired is not consistent. For example, the prophage-embedded fosSL is considered intrinsic while other prophage loci are considered acquired. The reasoning for this exception is not clear - as there is no evidence cited for the stability of the chromosomal integration in S.lugdunensis. This inconsistency weakens the proposed

classification framework. Please clarify and apply a consistent criterion throughout.
Please consider adding the limitations of this study in the discussion.

MINOR comments:
LINE-95: Please include genome QC metrics (completeness, contamination, assembly stats) in the supplementary data. Clarify whether all genomes were complete assemblies and whether plasmid data were available.
LINE-97: Include accession IDs for the reference gene sequences used.
Include software versions and citations for tBLASTn and IQ-TREE.
LINE-142: Consider adding a table listing all chosen reference genomes. Clarify what these were used for. Phylogenetic analysis typically does not require a single reference genome unless reference-based alignment was performed.
LINE-152: Include the strain name and sequence type for the laboratory-preserved strains.
LINE-231: Report both the number and percentage of genomes carrying each fos allele.
LINE-239: Since two distinct fosSA alleles were detected, the statement that fosSA is clonal across the species is inaccurate. Please rephrase to indicate clonality within specific sequence types.
Fig2A The brown bar on the right is unlabeled - presumably indicating absence of fos genes. Please label clearly.
LINE-253: The text references Figure 2F, which appears to be missing.
LINE-293: The term "host range" may be misleading, as it could be interpreted as the host range of Staphylococcus itself. Consider rephrasing.
LINE 366-368: This sentence is confusing—it currently implies that the subclassification of "acquired" genes depends on sequence variation among intrinsic alleles. Please clarify the intended meaning.
LINE-387- Add citations to support prior findings mentioned.

Reviewer #4

(Remarks to the Author)
The authors present a comprehensive study detailing the phylogenetic distribution of the major fosfomycin resistance determinant. Fosfomycin is getting increased attention as potential alternative antibiotic to treat staphylococcal infections as the pipeline for new antibiotics remains empty and resistance levels in S. aureus in certain parts of the world are alarmingly high.
The authors use robust and established in silico methods analysing a sizable dataset of staphylococcal genomes. They back up their computational findings with a limited in vitro approach to validate the identified Fosfomycin resistance determinants.
The findings are surely of interest to a distinct group of scientists as it cleans up and harmonizes a lot of the existing nomenclature and taxonomy of fosfomycin resistance determinants, which is at the moment certainly confusing. However, I doubt that the work is interest to a broader scientific audience as the manuscript is largely descriptive and does not provide novel insights into the mechanism of fosfomycin resistance, its clinical implications and the mechanisms of dissemination among staphylococci.
Major comments:
The authors identify novel versions of the fos gene and test them by orthologue expression in S. aureus RN4220. Here they observe drastic differences in the MIC against Fosfomycin whereas the core catalytic motif seems to be conserved (Fig. 1B). The authors should provide a mechanistic model, potentially guided by structure predictions and the creation to mutant variants, for the strong phenotypic differences in these variants.
Additionally rather than relying on only MIC analysis the authors should use bacterial killing assays to investigate the effects of different fos variants.
The authors claim that they harmonize the existing nomenclature of fos loci. I think it would be helpful for the interested readership if some sort of summary in a table or list is provided featuring different names for the same loci and how they are named according to the new taxonomy.
The author discovered fos resistance determinants on prophage-like elements. The authors should try to acquire some of the strains harbouring theses phage's try to mobilize the elements by phage induction and subsequent transduction into other staphylococi? This experiment would strengthen the mechanistic relevance of the manuscript. The authors even speculate of the potential contribution of mobile fos loci to increased fosfomycin MICs. This hypothesis could be tested by experimental wet-lab approaches.

Minor Comments:
In general, the spelling of bacterial names is often wrong and not consistend. Often the space between genus (in the abbreviated form) and species is not present.
The Figures present very different colour pallets which are not harmonized. Figure 1 presents a different colour palette than Fig 2 and 4 for example. Colour should be used consistently for the same concepts/groups. For example, fosB is labelled in a grey colour in Fig 1A but appears in green/cyan in Fig 4A etc.
Also, the fond size varies a lot in the figures many labels for fos variants are not readable at all (Fig 3D, for example).
Comments on Figure 1: Element C should be placed in the supplementary information or be presented in another form. Element B could benefit from the projection of the conserved elements an a protein structure of the Fos resistance-factor (for example PDB model 4NAY).
Comments on Figure 3. Here the distribution of fos variants across staphylococcal clades is explained. However, this occurs in totally different plots (Sankey diagrams, dendograms or (absolute or relative) barplots). I suggest to harmonize these different plots.
Line 105: the authored should explain what method/alogrythm was used for sequence alignment.

Line 152: The authors should list what kind of strains were used as source for PCR amplification of the locus.
Line 167: The authors should specify which phage was used for transduction. The Phage should appear in the list for bacterial strains and viruses.
Line 175: Agar dilution method is less common or standardized than liquid broth dilution to measure MICs. Here the authors should detail the exact methodology to allow reproduction of the results.

Line 27 +96: Staphylococcus not in italics
Line 29: There is an unwarranted space in “18,839”
Line 60: Gram-positive
Line 61: Gram-negative
Line 73 + 76: S. aureus
Line 84: Staphylococci not in italics
Line 233: homologues in; S. Argenteus
Line 273: almost “all” currently
Line 288: fos in italics
Line 390: resistance
Line 394: distributed

Reviewer #5

(Remarks to the Author)
I co-reviewed this manuscript with one of the reviewers who provided the listed reports. This is part of the Nature Communications initiative to facilitate training in peer review and to provide appropriate recognition for Early Career Researchers who co-review manuscripts.

Version 1:

Reviewer comments:

Reviewer #1

(Remarks to the Author)
I am happy with the changes that were made in response to my comments.

Reviewer #2

(Remarks to the Author)
The authors have done a very good job addressing each of the reviewer comments.

However, I have an additional query about a new figure that has appeared - Figure 6
(i) in order to understand the effects of fosfomycin on growth and killing, growth controls for each strain without fosfomycin should be included.
I see that the empty vector without fosfomycin is included in Fig 6B but this is not intuitive at first glance as it is the only bar in the graph that has no fosfomycin. It should be made clearer that this is the growth control.

Reviewer #4

(Remarks to the Author)
I have nothing to add.

Reviewer #5

(Remarks to the Author)
I co-reviewed this manuscript with one of the reviewers who provided the listed reports. This is part of the Nature Communications initiative to facilitate training in peer review and to provide appropriate recognition for Early Career Researchers who co-review manuscripts.

Open Access This Peer Review File is licensed under a Creative Commons Attribution 4.0 International License, which permits use, sharing, adaptation, distribution and reproduction in any medium or format, as long as you give appropriate credit to the original author(s) and the source, provide a link to the Creative Commons license, and indicate if changes were made.
In cases where reviewers are anonymous, credit should be given to 'Anonymous Referee' and the source.
The images or other third party material in this Peer Review File are included in the article's Creative Commons license, unless indicated otherwise in a credit line to the material. If material is not included in the article's Creative Commons license and your intended use is not permitted by statutory regulation or exceeds the permitted use, you will need to obtain permission directly from the copyright holder.
To view a copy of this license, visit https://creativecommons.org/licenses/by/4.0/

Reviewer #1 (Remarks to the Author):

The manuscript addresses fosfomycin resistance in Staphylococci, the genes involved and their nomenclature. It identifies novel genes and demonstrates that they increase fosfomycin resistance. In general, it would be useful with more information about the putative mode of action of the different resistance genes and if that was taken into account when forming the resistance gene nomenclature. Also did you observe any correlations between predicted modes of action and gene locations? Furthermore the authors do not address if there is any effect of Fosfomycin resistance and B-lactam resistance which may be predicted based on the mode of action of Fosfomycin.

Response: We sincerely thank the reviewer for the insightful comments. As requested, we have clarified in the revised manuscript that the fosfomycin resistance genes characterized in this study encode fosfomycin-inactivating enzymes that catalyze the conjugation of fosfomycin with bacillithiol, thereby inactivating the drug. Despite sequence diversity among the variants we identified, all genes retain the conserved catalytic residues essential for this enzymatic activity, supporting the conclusion that they share a common mode of action.

With respect to nomenclature, the putative mode of action was considered during gene classification. Because the identified *fos* genes share this conserved fosfomycin-modifying mechanism, we did not assign names according to distinct resistance mechanisms. Instead, nomenclature was guided by amino-acid identity, phylogenetic topology, genomic context and population-level distribution. We have clarified this rationale in the revised manuscript. At the same time, we agree that a shared core resistance mechanism does not exclude functional diversification among Fos variants. Indeed, the variable MICs observed in our heterologous expression assays suggest that differences in substrate accommodation, catalytic efficiency, expression level or host background may influence the strength of resistance. We have therefore added structural modelling and docking analyses and expanded the Discussion to address possible mechanistic differences among Fos variants.

Regarding the relationship between mode of action and gene location, our analyses did not indicate that different genomic locations correspond to fundamentally different resistance mechanisms. Intrinsic chromosomal genes, plasmid- or genomic-island-associated genes and prophage-associated genes all encode

Fos-family enzymes with conserved catalytic features. Thus, genomic location appears to reflect evolutionary origin and dissemination route more than a distinct mode of fosfomycin resistance. However, we have revised the manuscript to avoid overstating this point and now discuss that differences in expression context, host background and local protein architecture may contribute to the variable phenotypes observed among variants.

We also appreciate your comment regarding potential interactions between fosfomycin resistance and β-lactam susceptibility. Because fosfomycin targets MurA, an early step in peptidoglycan biosynthesis, we assessed whether *fos* gene expression influenced susceptibility to downstream cell wall-targeting antibiotics. We therefore expanded antimicrobial susceptibility testing to include the β-lactams cefoxitin and ceftobiprole across the heterologous expression constructs. No consistent β-lactam MIC changes were observed compared with the vector control, supporting the conclusion that these *fos* genes primarily affected fosfomycin susceptibility in this experimental system. In the LAC deletion background, β-lactam MICs were reduced after *fosSA* deletion, but this phenotype was not restored to the parental pattern by *fosSA* complementation. We therefore did not attribute these β-lactam changes to *fosSA* itself. Overall, these results support a specific contribution of *fos* genes to fosfomycin susceptibility in the experimental systems tested. These data and the corresponding clarification have been incorporated into the revised manuscript. We thank the reviewer again for helping us improve the clarity and precision of our manuscript.

Abstract: In general, the novelty and impact of the manuscript should be highlighted to a greater extent as there are other examples of resistance genes being present both in the core genome and present on various mobile genetic elements. Also the compound Fosfomycin should be introduced what it targets.

Response: We thank the reviewer for the constructive comments regarding the Abstract. In response, we have revised the Abstract to more clearly highlight the novelty and impact of our study. As the reviewer correctly noted, the presence of fosfomycin resistance genes in both core genomes and mobile genetic elements has been reported previously. However, prior work has not provided a systematic, genus-wide framework for classifying and naming *Staphylococcus* fosfomycin resistance genes. Our revised Abstract now emphasizes that this study establishes a

unified classification and nomenclature system for *fos* genes across clinically common *Staphylococcus* species, based on extensive genomic and phylogenetic analyses of 17,024 publicly available genomes. We also highlight that our work identifies a set of intrinsic *fos* genes embedded in the core genome, defines their taxonomic boundaries, and characterizes a series of previously unrecognized prophage-associated *fos* families.

Moreover, following the reviewer's suggestion, we have added a brief introduction of fosfomycin and its mode of action, noting that fosfomycin inhibits peptidoglycan precursor synthesis by targeting the enzyme MurA. This provides improved context for the biological relevance of the *fos* gene family.

We appreciate the reviewer's comments, which have substantially improved the clarity, completeness, and impact of the Abstract.

L 28: Change wording (the genes are not confused) and mention what exactly is confusing

Response: We thank the reviewer for this helpful comment. We agree that the previous wording was imprecise, because the genes themselves are not "confused"; rather, their annotation and nomenclature have been inconsistent. We have revised the wording throughout the manuscript to clarify this point. Specifically, the revised text now states that staphylococcal *fos* genes have been inconsistently annotated and lack a coherent taxonomic framework. A representative example is the broad use of the term *fosB* for diverse *fosB*-like homologues in *Staphylococcus*, including chromosomal species-associated genes such as *fosSA*, which have previously been referred to as *fosB* or *fosB*-like genes in some studies, despite being distinct from acquired plasmid-borne *fosB*. We have added this clarification and supporting references in the revised Introduction. This more accurately defines the nomenclature problem addressed by our study.

L 30: were these species selected for a reason?

Response: We appreciate the reviewer's question. We have clarified in the revised text that the 14 *Staphylococcus* species included in our analysis were selected because they are clinically common species with abundant publicly available genomes.

This provides both clinical relevance and sufficient genomic depth to support robust evolutionary and comparative analyses. The revised Abstract now explicitly describes these as “clinically common *Staphylococcus* species.”

L 38: MIC is not defined

Response: We thank the reviewer for noting this omission. We have now defined MIC (minimum inhibitory concentration) at its first appearance in the manuscript to ensure clarity and consistency.

L 43: Silent *fos* genes – does this mean that the genes are not providing resistance? The goes against the statement in line 39 about a 16 fold reduction in MIC

Response: We thank the reviewer for pointing out the ambiguity caused by our use of the term “silent.” Our intention was not to imply that these genes lack functional activity or do not confer resistance. Instead, we used “silent” to refer to the fact that the presence and distribution of many fosfomycin resistance (*fos*) genes across *Staphylococcus* species had previously been unrecognized or unannotated in public genomic datasets. Because this phrasing could be misunderstood, we have revised the wording in the Abstract and main text to avoid the term “silent” and to clearly state that our study reveals the previously unrecognized distribution of *fos* genes within the genus. This modification eliminates the ambiguity and ensures consistency with our experimental data showing that deletion of *fosSA* results in a 16-fold reduction in MIC.

Main text:

L 62: Expand on the description of resistance – what do you mean by defects?

Response: We thank the reviewer for this helpful comment. In the revised manuscript, we have clarified what is meant by “defects” in the glpT and uhpT transporters. Specifically, fosfomycin resistance mediated through these transport systems typically arises from missense mutations, frameshift mutations, or other disruptive alterations that lead to loss of transporter function, thereby preventing fosfomycin from entering the bacterial cell. This mechanism has been well documented in *Staphylococcus* and other bacteria, and we now describe these mutational inactivation events explicitly to avoid ambiguity.

L 68-78: Are these gene products providing resistance via *glpT* or *uhpT*?

Response: We thank the reviewer for the clarification request. The *fos* genes discussed in this section (*fosB*, *fosD*, and *fosY*) encode bacillithiol transferases that confer fosfomycin resistance through enzymatic inactivation of the drug. We have revised the text to explicitly describe this mechanism, which is distinct from transporter-mediated resistance, thereby clarifying the basis of resistance conferred by these gene products.

L 188: Provide an argument for selecting these species?

Response: We thank the reviewer for this question. The 14 *Staphylococcus* species included in this study were selected because they are commonly encountered in clinical settings and are well represented in public genome databases. This selection ensures both the clinical relevance of our analysis and sufficient genomic depth to support robust comparative and evolutionary analyses.

L 214-218: Mention if the type of resistance correlates with location (core, mobile, phage)

Response: We thank the reviewer for this comment. Our analyses demonstrate that all *fos* genes identified in this study encode fosfomycin-modifying enzymes with conserved catalytic features, regardless of whether they are located in the core chromosome, mobile genetic elements, or prophages. Thus, the resistance mechanism mediated by *fos* genes is uniform and independent of genomic location. However, while the catalytic mechanism itself appears conserved across genomic contexts, we cannot exclude the possibility that genomic location may influence gene expression level and thereby modulate the magnitude of the resistance phenotype, particularly through differences in copy number, promoter architecture, or local regulatory context associated with mobile elements. To avoid any potential ambiguity, we have now explicitly stated this point in the revised manuscript.

L. 213: Did you pay attention to putative mode of action when assigning gene names?

Response: We thank the reviewer for this insightful question. The putative mode of action was considered during gene classification and nomenclature. All *fos* genes identified in this study encode Fos-family fosfomycin-modifying enzymes and retain

conserved catalytic features of the VOC superfamily, supporting a shared core mechanism of fosfomycin inactivation. Therefore, gene names were not assigned according to different resistance mechanisms, as no evidence was found that distinct *fos* families mediate fosfomycin resistance through fundamentally different modes of action. Instead, nomenclature was guided by amino-acid identity, phylogenetic topology, genomic context and evolutionary distribution. We have also clarified that a shared resistance mechanism does not preclude broader biological functions. In particular, intrinsic *fos* genes may contribute to bacillithiol homeostasis, redox balance, bacterial fitness or other stress-adaptation processes beyond antibiotic detoxification. This point has been added to the revised Discussion as an important direction for future functional studies.

L278-279: with the report here or prior?

Response: We thank the reviewer for requesting clarification. The identification of *fosSL*, its localization within a prophage-like genomic island, and its high prevalence and conserved local genomic context within the analysed *S. lugdunensis* are all novel findings reported in this study, based on our genome-wide analyses. We have revised the text to explicitly indicate that these observations originate from the present work rather than from prior reports.

L. 327: mention if they are cloned with their own promoter or common heterologous promoters

Response: Thank you for this helpful suggestion. All *fos* genes were expressed under the control of the vector-encoded promoter in pTSSCm-pcap rather than their native promoters. This ensured a uniform transcriptional context across constructs, allowing direct comparison of their contributions to fosfomycin susceptibility. This information has now been clarified in the revised manuscript

L. 335-341: Given that Fosfomycin targets the UDP-N-acetylglucosamine-3-enolpyruvyltransferase there could be an interplay with B-lactam susceptibility – this should be tested for all your constructs.

Response: We thank the reviewer for this insightful mechanistic suggestion. As fosfomycin targets MurA, an early step in peptidoglycan biosynthesis, we evaluated

whether expression of *fos* genes influences susceptibility to downstream cell wall-targeting β-lactams.We therefore assessed susceptibility to cefoxitin and ceftobiprole across all heterologous expression constructs. No measurable changes in MICs were observed compared to the vector control, indicating that *fos* gene expression does not alter β-lactam susceptibility in this experimental system. These data have now been incorporated into the revised manuscript.

In the LAC background, deletion of *fosSA* resulted in a modest change in cefoxitin MIC; however, this phenotype was not restored by *fosSA* complementation. We have clarified in the revised manuscript that this observation does not imply a functional role for *fosSA* in β-lactam susceptibility. The data specifically support the contribution of *fosSA* to fosfomycin resistance.

Reviewer #2 (Remarks to the Author):

Happy to have the opportunity to review the manuscript entitled "Decoding a silent reservoir of fosfomycin resistance genes in *Staphylococcus*: A genomic and evolutionary study".

Title. To begin, I do not quite understand the title. Why is it a "silent reservoir"? The genes seem to provide some low/moderate level resistance to fosfomycin and they have other physiological functions within the cell. Why silent?

Response: We thank the reviewer for pointing out this ambiguity. We agree that the term “silent” could be misleading, because the genes characterized in this study are not functionally silent; several of them clearly affected fosfomycin susceptibility, and some intrinsic *fos* genes may also have broader physiological roles. Our original intention was to indicate that many *fos* homologues in staphylococci had been previously underrecognized or inconsistently annotated in public genomic datasets. We have therefore revised the title to “Decoding an underrecognized fosfomycin resistance gene repertoire in Staphylococcus: a genomic and evolutionary study.”

Abstract. As above "this work uncovers a previously hidden ... reservoir of silent *fos* genes..." were they hidden because they only have a small impact of fosfomycin susceptibility? Please try to clarify.

Response: We thank the reviewer for this helpful comment. We agree that the previous wording “hidden” and “silent” could be misleading, and this phrase has been removed from the revised Abstract. We did not intend to imply that these genes were hidden because they have only a small effect on fosfomycin susceptibility. In fact, our cloning and susceptibility assays showed that different *fos* genes produced variable effects on fosfomycin MICs, ranging from minimal changes to substantial increases. Our intended meaning was that many staphylococcal *fos* homologues had previously been underrecognized because earlier studies often used overlapping *fosB*-like annotations and because a comprehensive genome-wide screening and classification framework had not been applied. We have revised the Abstract and main text to clarify this point and now describe these genes as a previously underrecognized *fos* reservoir rather than as “hidden” or “silent.”

Intro. The intro should give some introduction to the function of the enzyme coded by *fos* genes. What does it do?

Response: We thank the reviewer for this helpful suggestion. We have revised the Introduction to include a clearer description of the function of enzymes encoded by *fos* genes. Specifically, we now state that *fos* genes encode fosfomycin-modifying enzymes, such as bacillithiol transferases in staphylococci, which confer resistance by enzymatically inactivating fosfomycin. This mechanism is distinct from transporter-mediated resistance caused by loss-of-function mutations in *glpT* or *uhpT*, which reduce fosfomycin uptake. This clarification has been incorporated into the revised Introduction.

Line 56 - I think these two references should be discussed with more care/caution. There was no statistically significant improvement compared to monotherapy when fosfomycin was included in those studies.

Response: We thank the reviewer for this important clarification. We agree that the clinical evidence for fosfomycin-containing combination therapy should be presented with appropriate nuance. In the revised Introduction, we have avoided implying a uniform clinical benefit across all settings. Instead, we now state that fosfomycin has been evaluated as an adjunctive or combination option for severe *S. aureus* infections, and that recent trial-based and pooled analyses suggest its clinical value may be context-dependent, varying with infection complexity, patient subphenotype and treatment setting. This balanced wording preserves the clinical rationale for studying fosfomycin resistance determinants while more accurately reflecting the available evidence.

Line 68 - the sentence is not clear to me, "with amino acid identities ranging from 62% to 80%," compared to what?

Response: We thank the reviewer for pointing out this ambiguity. This sentence referred to the amino acid identities among the series of chromosomal *fosA* genes identified in the cited study, rather than their identity to a single reference sequence. We have revised the sentence to clarify that these *fosA* homologues shared 62–80% amino acid identity with one another.

Line 76 - "Notably, many researchers have failed to distinguish..." This warrants some type of reference/some evidence.

Response: We thank the reviewer for this careful suggestion. We agree that the original wording was too strong and should be supported by evidence. We have replaced "failed to distinguish" with a more neutral statement indicating that chromosomally encoded *fosSA*-like genes in *S. aureus* have not been consistently distinguished from acquired plasmid-borne *fosB* and have often been annotated or discussed under broad *fosB*-like names. We have also added relevant references to support this point and to clarify the need for standardized nomenclature.

Small point - Staphylococci is not the genus name, so it is not in italics, *Staphylococcus* is the genus name and should be in italics. Also, *fos*, indicative of a gene, should be consistently in italics.

Response: We have carefully checked the manuscript and standardized bacterial nomenclature and gene formatting throughout. Formal genus and species names are italicized, abbreviated genus names are followed by a space before the species epithet, common terms such as staphylococci are not italicized, and *fos* gene names are consistently italicized.

Materials methods. Why were only 14 species used? Without a rationale for exclusion I think more species should be included.

Response: We thank the reviewer for this important comment. The 14 *Staphylococcus* species were selected because they are commonly encountered in clinical settings or recognized as clinically relevant opportunistic pathogens, and because sufficient publicly available genome assemblies were available to support comparative genomic and phylogenetic analyses.

Provide refs for Snippy and iToL

Response: We thank the reviewer for this suggestion. In the revised manuscript, we have updated the species-level phylogenetic reconstruction workflow. The previous Snippy-based reference-alignment approach has been replaced by a Prokka/Panaroo/IQ-TREE workflow. Specifically, genomes were annotated using Prokka, core-gene alignments were generated using Panaroo, and

maximum-likelihood phylogenies were reconstructed using IQ-TREE. We have added the corresponding software versions and references for Prokka, Panaroo, IQ-TREE and iTOL in the revised Methods section

.

Does RN4220 have a *fos* gene?

Response: We thank the reviewer for raising this point. *S. aureus* RN4220 contains an endogenous intrinsic *fosSA* homologue. However, all heterologous expression experiments were performed in the same RN4220 background and compared directly with the empty-vector control RN4220/pT. Therefore, the effect of the endogenous *fosSA* background was controlled for, and the observed MIC changes reflect the additional contribution of the cloned *fos* genes under the vector-encoded promoter.

We also note that RN4220 remained fosfomycin-susceptible despite carrying endogenous *fosSA*. This is consistent with our interpretation that intrinsic *fos* genes do not necessarily confer clinical resistance at baseline in all genetic backgrounds. Instead, they may contribute to baseline MIC shifts or enhance bacterial adaptation under fosfomycin pressure, as discussed in the revised Discussion.

What media was used for the MIC - was it according to CLSI/EUCAST guidance?

Response: We have expanded the antimicrobial susceptibility testing section to specify that fosfomycin MICs were determined by agar dilution using Mueller–Hinton agar supplemented with glucose-6-phosphate, consistent with CLSI-recommended fosfomycin testing methodology. Fosfomycin MICs were interpreted according to EUCAST criteria, whereas other antibiotics were interpreted according to CLSI breakpoints.

Results - can you relate phylogeny of the intrinsic *fos* genes with general phylogeny of the species. I guess it would be similar?

Response: We thank the reviewer for this insightful suggestion. We agree that comparing intrinsic *fos* relationships with host phylogeny is useful for interpreting their evolutionary background. We therefore examined this point by comparing a representative 16S rRNA-based species tree with the phylogeny of representative intrinsic Fos proteins. The comparison showed partial concordance: for example, *fosSA/fosSA2* clustered together, consistent with the close relationship between *S.*

aureus and *S. argenteus*, and *fosSC/fosSCp* clustered together, consistent with the relatedness of *S. capitis* and *S. caprae*. However, the two trees were not fully congruent; for example, *fosSL* clustered closer to *fosSC/fosSCp* in the *fos* tree, whereas *S. lugdunensis* was not closely grouped with *S. capitis* or *S. caprae* in the 16S rRNA tree.

Because this comparison was only partially concordant and a 16S rRNA-based tree has limited resolution for formal cophylogenetic inference, we did not include this analysis as a main result or supplementary figure. Instead, we used it to guide a more cautious interpretation in the revised manuscript. We now describe intrinsic *fos* genes as showing species-, ST- or clade-associated distribution patterns, rather than claiming strict co-evolution between intrinsic *fos* genes and host species.

Line 226-232 and Figure 2A - Can a comment be made on the frequency of those ST in, for example, hospital environments, lifestock, etc environments? Could a particular *fos* allele be associated with "successful/prominent lineages" of *S. aureus*?

Response: We thank the reviewer for this insightful suggestion. We have revised the Results to comment on the relationship between *fosSA* distribution and major *S. aureus* lineages. In our dataset, the most apparent pattern was not a simple ecological niche-specific association of a particular *fosSA* allele, but rather ST-associated presence/absence and allele composition. Several hospital-associated epidemic lineages, such as ST5 and ST239, frequently carried *fosSA*, predominantly *fosSA.1*. Some community-associated lineages, such as ST8, also carried *fosSA.1*, whereas other community-associated lineages, including ST1 and ST59, lacked detectable *fosSA*. The livestock-associated lineage ST398 also lacked detectable *fosSA* in the analysed genomes. We have revised the text to clarify that *fosSA* distribution is associated with prominent clonal backgrounds, but does not appear to correspond to a single ecological niche category. We appreciate this comment, as it prompted us to consider the clonal heterogeneity of *fosSA* carriage from a broader biological perspective. Whether the lineage-specific presence or absence of *fosSA* reflects additional physiological or adaptive roles beyond fosfomycin resistance is an interesting question that warrants future investigation.

To help interpret the figure, is the relative size of each band on the left of Figure 2A

indicative of the relative proportion of each ST in your analysis? I think could be mentioned in the legend.

Response: We thank the reviewer for this suggestion. The original Sankey plot in Figure 2A has been replaced with an ST-based stacked bar plot in the revised figure. Thus, relative band size is no longer applicable. We have revised the legend to clarify that each bar represents a major ST and that stacked segments indicate the proportion of genomes within that ST carrying each intrinsic *fos* allele category.

Figure 2 - It is odd that the same type of information across the 5 panels is presented in 5 different ways. Makes it difficult to compare across the species. Why are only 5 of the 14 species assessed in this type of analysis? At least a comment should be made as to why the others did not get the same assessment.

Response: We thank the reviewer for this helpful comment. We have revised Figure 2 to improve consistency and readability. Because species-level prevalence of *fos* genes is already summarized in Table 1, the revised Figure 2 now focuses specifically on within-species clone- or clade-associated distribution of intrinsic *fos* alleles. For *S. aureus*, *S. epidermidis* and *S. argenteus*, where ST-based population frameworks were available, allele distributions are shown using consistent stacked bar plots. For *S. capitis*, because the number of available genomes was more limited and the aim was to directly show the clade-associated distribution of *fosSC* in the whole-genome background, we presented *fosSC* carriage on a core-genome phylogeny. The *S. caprae* phylogeny has been moved to the Supplementary Information to reduce complexity in the main figure. For *S. lugdunensis*, the currently identified *fosSL* alleles were highly conserved and showed little allele-level variation; therefore, this species was not included as a separate figure panel and is instead summarized in Table 1. We have also clarified in the figure legend that colours indicate allele categories within each panel, ND indicates no corresponding intrinsic *fos* allele detected, and asterisks denote alleles containing premature stop codons.

Line 273 - there is a word missing in this sentence (possibly "...almost ALL currently...")

Response: We thank the reviewer for pointing this out. The sentence has been corrected in the revised manuscript.

Line 322 - what evidence do you have that the genes are cryptic? For example, *fosSC* is not a cryptic gene. When it is present, the MIC for fosfomycin is very high https://www.sciencedirect.com/science/article/pii/S0924857924000803. Not sure what is cryptic about that.

Response: We thank the reviewer for pointing this out and fully agree that "cryptic" was not an appropriate term here. In particular, *fosSC* is not cryptic, as it is a functional fosfomycin resistance determinant and, as the reviewer noted, can be associated with high fosfomycin MICs when present. Our intention was not to imply a lack of function, but rather to describe the fact that many *fos* homologues in *Staphylococcus* have remained underrecognized or inconsistently annotated in genomic databases and in the literature. To avoid this misleading implication, we have removed "cryptic" from the revised manuscript and replaced it with more precise wording, including "previously underrecognized," "previously unrecognized," or "representative *fos* genes," depending on the context. The corresponding section title and text have been revised accordingly..

The fosfomycin MICs for Table 2 should be the median of multiple measurements (with range included, if appropriate). This can help to reduce the potential impact of intrinsic error associated with the agar dilution method.

Response: We thank the reviewer for this helpful suggestion. Fosfomycin MIC testing was performed in independent measurements, and the revised table now reports the median MIC values with ranges where applicable.

Reviewer #3 (Remarks to the Author):

This manuscript presents a comprehensive genomic analysis of 18,839 *Staphylococcus* genomes, uncovering a previously unrecognized diversity of *fos* genes associated with fosfomycin resistance. The authors introduce a novel identification and classification framework for *fos* gene families and report five intrinsic chromosomal determinants alongside eight novel prophage-associated families, with variable levels of resistance through distinct evolutionary trajectories and horizontal gene transfer mechanisms that threaten the clinical utility of fosfomycin.

Overall, this is a useful and well-structured study. However, several important clarifications and methodological details are needed before the conclusions can be fully supported.

MAJOR comments:

The observed diversity of *fos* gene families appears highly dependent on the underlying sampling distribution of genomes. The authors apply a retention criterion of alleles present in ≥3 genomes; however, the sampling across *Staphylococcus* species is uneven. This could inflate signals from overrepresented species and lineages while filtering out rare determinants, leading to underestimation of novel gene families.

Please consider implementing normalization (e.g., subsampling or weighted inference) to mitigate sampling bias and clarify how uneven sampling may affect claims of diversity and ubiquity across species.

Response: We thank the reviewer for highlighting the potential impact of uneven sampling on the detection of *fos* diversity. In the original analysis, the criterion requiring candidate alleles to be present in ≥3 genomes was used as a conservative filter to reduce potential assembly artefacts or spurious matches. Following the reviewer's suggestion, and after applying additional genome-quality filtering, we re-analysed the dataset without this retention threshold. This allowed rare but genuine *fos* variants to be retained while relying on genome-quality metrics, sequence inspection and genomic-context verification to minimize false-positive calls.

We also refined the workflow for identifying and classifying novel *fos* genes. In the revised analysis, candidate homologues were evaluated not only by sequence similarity but also by phylogenetic placement, with bootstrap support used as an

additional criterion to assess the robustness of lineage assignment. This refinement led to a revision of the nomenclature for two prophage-associated *fos* lineages. The sequences originally labelled as *fosSΦB2* and *fosSΦF2* showed similarity to *fosSΦB* and *fosSΦF*, respectively, but did not cluster with these families with sufficient bootstrap support in the focused phylogenetic analysis. To avoid over-merging phylogenetically unsupported groups, we conservatively reclassified them as independent prophage-associated families, now designated *fosSΦI* and *fosSΦJ*.

To further verify the reliability of these rare prophage-associated *fos* candidates, we manually inspected the corresponding contigs. Candidates were retained only when the surrounding contig contained conserved staphylococcal flanking genes consistent with the host genome or recognizable prophage-associated modules, such as integrase, terminase, capsid, tail, primase or replication-associated genes. Candidates located on short contigs lacking informative flanking regions or showing inconsistent genomic context were excluded. These refinements have been incorporated into Supplementary Methods, figures and supplementary tables.

We also acknowledge that genome numbers are uneven across *Staphylococcus* species, reflecting the current structure of public genome databases rather than selective sampling by our study. To assess whether this imbalance affected the inferred distribution and classification of *fos* genes, we performed species-level subsampling analysis. For each species, up to 120 genomes were randomly selected, while all genomes were retained for species with fewer than 120 genomes. This procedure was repeated ten times. The subsampling analysis yielded consistent *fos* distribution patterns and did not alter the major classification framework. In particular, acquired *fos* genes remained infrequent in the highly sampled *S. aureus* population, whereas prophage-associated *fos* genes remained enriched in less-sampled species such as *S. saprophyticus* and *S. cohnii*. These results suggest that the major diversity and distribution patterns observed in this study were robust to our subsampling scheme, although rare lineages and prevalence estimates remain influenced by current public genome availability.

We have added the subsampling analysis to the Methods and included the results in the Supplementary Table 5. We also revised the Results and Discussion to clarify that prevalence estimates should be interpreted in light of the uneven representation of public genomes.

The methods section lacks details on the quality control of the 18,839 genomes obtained from NCBI. Were all genomes complete assemblies? Were plasmid sequences available for all isolates? Since assembly completeness and plasmid representation can directly influence the detection of *fos* loci, these details are essential. Please include metrics such as completeness, contamination, and assembly statistics in the supplementary materials.

It is critical to ensure that the 164 identified *fos* sequences originate from *Staphylococcus* assemblies and not from contaminants or misassembled contigs. Please describe any QC steps or filtering criteria used to verify the authenticity of these sequences.

Response: We thank the reviewer for emphasizing the importance of genome quality control. All 18,839 genomes were retrieved from the NCBI RefSeq database, including assemblies annotated at the levels of Complete Genome, Scaffold, and Contig, to ensure comprehensive representation of both chromosomal and mobile genetic elements.

Following the reviewer's suggestion, we applied additional quality control filtering to minimize potential artefacts. Specifically, genomes were retained only if they met the following criteria: CheckM completeness ≥95%, contamination ≤3%, assembly contig number <200, and contig N50 >50 kb (with adjusted thresholds for *S. hominis* due to assembly characteristics). These metrics have now been included in the supplementary materials.

Although plasmid and chromosomal contigs were not analyzed separately, all identified *fos* loci were manually verified based on their genomic context. In each case, flanking genes and sequence composition were consistent with staphylococcal genomic environments, supporting their authenticity and reducing the likelihood of contamination or misassembly.

These additional quality control steps have been clarified in the revised Methods section.

Because of the highly uneven number of genomes analyzed across species, it is important to consistently include the total number of genomes (n) whenever percentages are reported throughout the Results section.

Response: We agree with the reviewer. We have revised the Results section and Table 1 to consistently report both the number of positive genomes and the total number of genomes whenever percentages are presented.

The categorization of intrinsic v/s acquired is not consistent. For example, the prophage-embedded *fosSL* is considered intrinsic while other prophage loci are considered acquired. The reasoning for this exception is not clear - as there is no evidence cited for the stability of the chromosomal integration in *S. lugdunensis*. This inconsistency weakens the proposed classification framework. Please clarify and apply a consistent criterion throughout.

Response: We thank the reviewer for this important comment. We agree that our original wording did not make the classification criteria sufficiently clear and may therefore have created the impression of inconsistency. In the revised manuscript, we now state explicitly that the intrinsic/acquired assignment was based on a combined operational framework including prevalence within a species or major lineage, phylogenetic distribution pattern, conservation of local genomic context, and evidence of mobility-associated patchy occurrence. Prophage localization alone was not used as a defining criterion.

Under this framework, *fosSL* was treated as an atypical intrinsic *fos* determinant in *S. lugdunensis* because it was detected in >97% of the analysed genomes and was associated with a conserved local genomic context across the available dataset, rather than the sporadic and variable prophage-associated patterns observed for the acquired *fosSΦ* families. Although *fosSL* is embedded in a prophage-like region, its near species-wide distribution in the current dataset is more consistent with a lineage-associated, stably maintained element than with a recently or frequently mobile determinant.

By contrast, *fosSΦB* in *S. saprophyticus* and *fosSΦD* in *S. cohnii*, despite relatively high prevalence in their respective datasets, showed patchy distributions within species phylogenies, with presence/absence variation among related subclades, supporting their interpretation as acquired prophage-associated determinants rather than intrinsic genes.

We also acknowledge that these inferences are based on currently available genomes and that limited sampling in some species may affect resolution. We have

therefore clarified the operational criteria in the revised manuscript and added this limitation to the Discussion.

Please consider adding the limitations of this study in the discussion.

Response: We thank the reviewer for this suggestion. We have added a dedicated limitations paragraph to the Discussion. We now acknowledge the uneven representation of public genomes, the potential limitations of draft assemblies for reconstructing plasmids and prophages, the need for experimental validation of prophage mobility, and the limitations of heterologous expression and predictive structural modelling.

MINOR comments:

LINE-95: Please include genome QC metrics (completeness, contamination, assembly stats) in the supplementary data. Clarify whether all genomes were complete assemblies and whether plasmid data were available.

Response: We thank the reviewer for this helpful suggestion. We have added genome-quality metrics, including CheckM completeness, CheckM contamination, contig number and contig N50, to the supplementary data. We also clarified in the Methods that the dataset included NCBI RefSeq assemblies at different assembly levels, including complete genomes, scaffolds and contigs, rather than only complete genomes. Because plasmids are not always resolved as separate replicons in draft assemblies, plasmid and chromosomal contigs were not analysed separately; instead, *fos* loci were assigned based on their genomic context and flanking regions.

LINE-97: Include accession IDs for the reference gene sequences used.
Include software versions and citations for tBLASTn and IQ-TREE.

Response: We thank the reviewer for this helpful suggestion. We have added the accession IDs of the reference *fos* sequences used for homologue searches to Supplementary Table 1. We have also added the software versions and relevant citations for BLAST and IQ-TREE in the revised Methods section.

LINE-142: Consider adding a table listing all chosen reference genomes. Clarify what these were used for. Phylogenetic analysis typically does not require a single reference genome unless reference-based alignment was performed.

Response: We thank the reviewer for this helpful suggestion. We agree that the previous description could be misleading because a single reference genome is not required unless reference-based alignment is used. To avoid this ambiguity, we revised the phylogenetic reconstruction workflow. In the revised analysis, genomes of the relevant *Staphylococcus* species were annotated using Prokka, core-gene alignment was generated using Panaroo, and maximum-likelihood phylogenies were reconstructed using IQ-TREE. The resulting trees were midpoint-rooted before visualization. Because this revised workflow no longer relies on a single reference genome for phylogenetic reconstruction, a table of selected reference genomes for tree building is no longer applicable. Reference genomes were used only for comparative analysis of the genomic regions surrounding *fos* genes, and the accessions of all genomes used for genomic-context comparisons have been provided in the supplementary table. The Methods section has been revised accordingly.

LINE-152: Include the strain name and sequence type for the laboratory-preserved strains.

Response: We agree with the reviewer. We have added the strain names and sequence types of the laboratory-preserved strains used as PCR templates to Supplementary Table 4 and clarified this in the Methods.

LINE-231: Report both the number and percentage of genomes carrying each *fos* allele.

Response: Thank you for the suggestion. We have revised the Results section and Table 1 to report both the number of positive genomes and the total number of genomes whenever percentages are presented.

LINE-239: Since two distinct *fosSA* alleles were detected, the statement that *fosSA* is clonal across the species is inaccurate. Please rephrase to indicate clonality within specific sequence types.

Response: We thank the reviewer for this important clarification. We have revised the text to avoid implying that *fosSA* is clonal across the entire species. The revised Results now describe *fosSA* and *fosSA2* as showing ST-associated distribution patterns, involving both presence/absence and allele composition. Specifically, some STs frequently carry these genes whereas others lack detectable homologues, and

among positive STs, particular alleles are often dominant. This wording more accurately reflects the patterns shown in revised Figure 2.

Fig2A The brown bar on the right is unlabeled - presumably indicating absence of *fos* genes. Please label clearly.

LINE-253: The text references Figure 2F, which appears to be missing.

Response: We thank the reviewer for pointing this out. We have revised Figure 2 and its legend to clarify the meaning of the absence category. We also checked and corrected the figure citations in the main text to ensure that all panel references correspond to the revised figure layout.

LINE-293: The term "host range" may be misleading, as it could be interpreted as the host range of *Staphylococcus* itself. Consider rephrasing.

Response: We thank the reviewer for this helpful suggestion. We have replaced "host range" with "detected across the widest range of *Staphylococcus* species" to avoid ambiguity.

LINE 366-368: This sentence is confusing—it currently implies that the subclassification of "acquired" genes depends on sequence variation among intrinsic alleles. Please clarify the intended meaning.

Response: We thank the reviewer for pointing out this ambiguity. In the revised manuscript, this confusing sentence has been removed. We have rewritten the relevant Discussion section to clarify that intrinsic and acquired *fos* genes were classified using a unified framework integrating amino-acid identity, phylogenetic topology, genomic context and population-level distribution. The subclassification of acquired genes is therefore not based on sequence variation among intrinsic alleles, but on the combined evidence of sequence divergence, phylogenetic clustering and genomic context within the corresponding acquired or prophage-associated groups.

LINE-387- Add citations to support prior findings mentioned.

Response: We thank the reviewer for this suggestion. The corresponding section has been substantially revised and shortened in the revised Discussion. We have also added citations where prior findings are mentioned to ensure that the statements are appropriately supported.

Reviewer #4 (Remarks to the Author):

The authors present a comprehensive study detailing the phylogenetic distribution of the major fosfomycin resistance determinant. Fosfomycin is getting increased attention as potential alternative antibiotic to treat staphylococcal infections as the pipeline for new antibiotics remains empty and resistance levels in *S. aureus* in certain parts of the world are alarmingly high.

The authors use robust and established in silico methods analysing a sizable dataset of staphylococcal genomes. They back up their computational findings with a limited in vitro approach to validate the identified Fosfomycin resistance determinants.

The findings are surely of interest to a distinct group of scientists as it cleans up and harmonizes a lot of the existing nomenclature and taxonomy of fosfomycin resistance determinants, which is at the moment certainly confusing. However, I doubt that the work is interest to a broader scientific audience as the manuscript is largely descriptive and does not provide novel insights into the mechanism of fosfomycin resistance, its clinical implications and the mechanisms of dissemination among staphylococci.

Response: We sincerely thank the reviewer for the thoughtful and constructive assessment. We appreciate the reviewer's recognition that harmonizing the nomenclature and taxonomy of staphylococcal *fos* determinants is valuable, and we fully understand the concern that the original manuscript was overly descriptive and did not sufficiently develop the mechanistic, clinical and dissemination-related implications of the findings. In response, we have substantially revised the manuscript. We added structural modelling and fosfomycin docking analyses to provide a mechanistic framework for the variable resistance phenotypes among Fos variants, and we expanded phenotypic validation beyond MICs by adding time-kill and growth-curve assays under fosfomycin exposure. We also tested β-lactams to assess potential interplay with downstream cell wall-targeting antibiotics. In addition, we extensively revised the Discussion to emphasize the clinical relevance of intrinsic *fos* genes, the role of coagulase-negative staphylococci as potential reservoirs, the potential interspecies dissemination of prophage-associated *fosSΦ* genes, and the limitations of our genomic and functional analyses.

Beyond their relevance to clinical fosfomycin resistance, our findings also highlight a broader biological question: the widespread conservation of several

intrinsic *fos* genes suggests that their roles may not be limited to antibiotic inactivation alone, and that the biological functions of *fos* genes themselves warrant further investigation. We believe these revisions strengthen the broader significance of the study while preserving its central contribution: a reproducible framework for identifying, classifying and naming *fos* genes in staphylococci.

Major comments:

The authors identify novel versions of the *fos* gene and test them by orthologue expression in *S. aureus* RN4220. Here they observe drastic differences in the MIC against Fosfomycin whereas the core catalytic motif seems to be conserved (Fig. 1B). The authors should provide a mechanistic model, potentially guided by structure predictions and the creation to mutant variants, for the strong phenotypic differences in these variants.

Additionally rather than relying on only MIC analysis the authors should use bacterial killing assays to investigate the effects of different *fos* variants.

Response: We thank you for this important and constructive suggestion. We agree that the original manuscript did not sufficiently explain the strong phenotypic differences observed among Fos variants. In the revised manuscript, we have expanded the functional validation and added predictive structural analysis to provide a more explicit mechanistic framework, while avoiding overinterpretation of docking-based predictions.

First, we expanded the phenotypic analysis beyond MIC measurements. Representative low-, intermediate- and high-resistance variants were selected for time-kill assays and growth-curve analyses under fosfomycin exposure. These experiments showed that the MIC differences translated into distinct biological behaviours: strains expressing low-resistance variants showed killing profiles similar to the vector control, whereas strains expressing intermediate- and high-resistance variants displayed partial or pronounced survival and regrowth. These data provide additional functional evidence that the identified *fos* variants are not equivalent in their protective capacity under fosfomycin pressure.

Meanwhile, we performed AlphaFold Server-based structural prediction and CB-Dock2 fosfomycin docking to provide structural context for this functional heterogeneity. The analysis showed that representative Fos variants retained the conserved VOC scaffold and recurrent pocket-associated residues, supporting a

shared Fos-family catalytic framework. However, predicted docking scores did not correlate linearly with fosfomycin MICs, and variants with similar predicted binding energies sometimes displayed markedly different resistance phenotypes. We therefore interpreted the docking results qualitatively, focusing on conserved pocket features and possible differences in substrate accommodation, rather than treating docking scores as quantitative measures of enzymatic activity.

Based on these revised analyses, we now discuss a model in which Fos-mediated resistance is not determined simply by the presence of conserved catalytic motifs or predicted fosfomycin binding energy. Instead, resistance magnitude may depend on additional factors, including productive substrate positioning, accommodation of the physiological thiol donor, catalytic turnover, protein dynamics and host-context-dependent functional performance. Because the tested *fos* genes originated from different staphylococcal species but were expressed in a common *S. aureus* RN4220 background, native host-specific expression or biochemical context may also influence their apparent activity. We have revised the Results and Discussion to present this interpretation explicitly and cautiously.

We agree that residue-level mutagenesis and purified-enzyme kinetic assays would be valuable for defining the detailed biochemical basis of these differences. However, because the primary focus of this study is the genomic identification, classification and evolutionary analysis of staphylococcal *fos* genes, such detailed enzymological dissection is beyond the scope of the current work. We now acknowledge this limitation and identify mutagenesis, enzyme kinetics and native-host validation as important future directions.

The authors claim that they harmonize the existing nomenclature of *fos* loci. I think it would be helpful for the interested readership if some sort of summary in a table or list is provided featuring different names for the same loci and how they are named according to the new taxonomy.

Response: We thank the reviewer for this helpful suggestion. We agree that a cross-reference table would improve the accessibility and practical utility of the proposed nomenclature. We have therefore added a supplementary table summarizing previously used names and database annotations, their sources, representative accessions, the corresponding names proposed or retained in this study, genomic context, and the rationale for reassignment. This table includes chromosomal

fosB-like annotations reclassified as species-associated intrinsic genes such as *fosSA* and *fosSE*, genes previously identified by our group whose names are retained, including *fosSC* and *fosY*, established acquired genes such as *fosB* and *fosD*, and newly defined prophage-associated families *fosSΦA–J*. We have also added a sentence in the Results section directing readers to this table.

The author discovered *fos* resistance determinants on prophage-like elements. The authors should try to acquire some of the strains harbouring theses phage's try to mobilize the elements by phage induction and subsequent transduction into other staphylococci? This experiment would strengthen the mechanistic relevance of the manuscript. The authors even speculate of the potential contribution of mobile *fos* loci to increased fosfomycin MICs. This hypothesis could be tested by experimental wet-lab approaches.

Response: We thank for this valuable suggestion.

We agree that experimental demonstration of prophage mobilization would provide important functional insight. However, the primary aim of this study was to characterize the diversity and genomic context of *fos* genes rather than to investigate prophage transfer experimentally. Such induction and transduction assays would require access to specific donor strains and optimized phage systems, which were not available within the scope of this work.

We have therefore limited our conclusions to the potential mobility inferred from conserved phage-related features. Experimental validation of prophage-mediated transfer represents an important direction for future investigation.

Minor Comments:

In general, the spelling of bacterial names is often wrong and not consistend. Often the space between genus (in the abbreviated form) and species is not present.

Response: We thank the reviewer for pointing this out, and we apologize for these careless formatting inconsistencies. We have carefully checked the manuscript and corrected the formatting and spelling of bacterial names throughout.

The Figures present very different colour pallets which are not harmonized. Figure 1 presents a different colour palette than Fig 2 and 4 for example. Colour should be used consistently for the same concepts/groups. For example, *fosB* is labelled in a

grey colour in Fig 1A but appears in green/cyan in Fig 4A etc.
Also, the fond size varies a lot in the figures many labels for *fos* variants are not readable at all (Fig 3D, for example).

Response: We thank the reviewer for this helpful comment. We have revised the figures to improve consistency and readability. The colour scheme has been harmonized across the manuscript so that the same *fos* families or categories are represented consistently in different figures. In particular, the colours used for acquired *fos* genes in Fig. 4A have been aligned with the corresponding gene-family colours in Fig. 1A, including *fosB*, *fosD*, *fosY* and the prophage-associated *fosSΦ* group. We enlarged the *fos* variant labels in the phylogenetic panels and standardized font sizes, legends and colour schemes across panels. We also checked the genomic-context figures and enlarged key gene labels where necessary.

Comments on Figure 1: Element C should be placed in the supplementary information or be presented in another form. Element B could benefit from the projection of the conserved elements an a protein structure of the Fos resistance-factor (for example PDB model 4NAY).

Response: We thank the reviewer for these helpful suggestions. We have revised Figure 1 to improve clarity, readability and mechanistic interpretation. The colour scheme in Fig. 1A has been harmonized with the corresponding *fos* categories used in subsequent figures, with prophage-associated *fosSΦA–J* families grouped using a single colour. We also added a new structural panel in Fig. 1C, in which representative conserved pocket residues are mapped onto the AlphaFold-predicted FosSA structure with docked fosfomycin, linking the conserved sequence features in Fig. 1B to the predicted substrate-binding pocket. The alignment display, font sizes and labels have been improved, and the legend now clarifies that asterisks denote alleles containing premature stop codons. The previous pairwise identity matrix has been moved out of the main figure to improve clarity.

Comments on Figure 3. Here the distribution of *fos* variants across staphylococcal clades is explained. However, this occurs in totally different plots (Sankey diagrams, dendograms or (absolute or relative) barplots). I suggest to harmonize these different plots.

Response: We thank the reviewer for this helpful suggestion. We interpreted this comment as referring to the figure showing the within-species distribution of intrinsic *fos* variants across clonal or phylogenetic backgrounds, which is now presented as revised Figure 2. We have revised this figure to harmonize the presentation across species. In the revised figure, *S. aureus*, *S. epidermidis* and *S. argenteus* are shown using consistent ST-based stacked bar plots, as ST-based population frameworks were available for these species. For *S. capitis*, because the number of available genomes was more limited and the aim was to directly show the clade-associated distribution of *fosSC* in the whole-genome background, we presented *fosSC* carriage on a core-genome phylogeny. We also standardized the colour schemes, legends and font sizes across panels. The revised figure now more clearly conveys that intrinsic *fos* alleles are generally conserved within major sequence types or phylogenetic clades.

Line 105: the authored should explain what method/alogrythm was used for sequence alignment.

Response: We thank the reviewer for this helpful suggestion. We agree that the sequence-alignment algorithm used for subsequent sequence comparison was not sufficiently specified. In this study, candidate *fos* homologues were first identified by BLASTN using nucleotide reference sequences. The candidate nucleotide sequences were then translated into amino-acid sequences and aligned using MUSCLE implemented in Geneious Prime. We have revised the Methods section to clarify that this amino-acid alignment was used for conserved-motif inspection, pairwise amino-acid identity comparison and downstream phylogenetic reconstruction.

Line 152: The authors should list what kind of strains were used as source for PCR amplification of the locus.

Line 167: The authors should specify which phage was used for transduction. The Phage should appear in the list for bacterial strains and viruses.

Response: We thank the reviewer for these helpful suggestions. To improve methodological clarity and reproducibility, we have added a supplementary table listing all bacterial strains, bacteriophages, plasmids and gene sources used in this study.

Line 175: Agar dilution method is less common or standardized than liquid broth dilution to measure MICs. Here the authors should detail the exact methodology to allow reproduction of the results.

Response: We thank the reviewer for requesting additional methodological detail. Fosfomycin MIC testing was performed using the agar dilution method because agar dilution is the CLSI-recommended reference method for fosfomycin susceptibility testing, with Mueller–Hinton agar supplemented with glucose-6-phosphate. We have now clarified this point in the revised Methods section and expanded the protocol to include the medium, glucose-6-phosphate supplementation, inoculum preparation, incubation conditions, replicate testing and quality-control strain. Susceptibility testing for non-fosfomycin antibiotics was performed by broth microdilution according to CLSI recommendations.

Line 27 +96: *Staphylococcus* not in italics

Line 29: There is an unwarranted space in “18,839”

Line 60: Gram-positive

Line 61: Gram-negative

Line 73 + 76: *S. aureus*

Line 84: Staphylococci not in italics

Line 233: homologues in; *S. Argenteus*

Line 273: almost “all” currently

Line 288: *fos* in italics

Line 390: resistance

Line 394: distributed

Response: We thank the reviewer for carefully noting these typographical and formatting issues, and we apologize for the careless inconsistencies. We have corrected all of the indicated items in the revised manuscript

Reviewer #5 (Remarks to the Author):

I co-reviewed this manuscript with one of the reviewers who provided the listed reports. This is part of the Nature Communications initiative to facilitate training in peer review and to provide appropriate recognition for Early Career Researchers who co-review manuscripts.

Response: We thank you for co-reviewing our manuscript and for contributing to the peer-review process. As no specific scientific comments were raised, no further response is required.

Reviewer #1:
I am happy with the changes that were made in response to my comments.

Response: We thank the reviewer for the positive assessment of our revised manuscript.

Reviewer #2:
The authors have done a very good job addressing each of the reviewer comments.
However, I have an additional query about a new figure that has appeared - Figure 6
(i) in order to understand the effects of fosfomycin on growth and killing, growth controls for each strain without fosfomycin should be included.
I see that the empty vector without fosfomycin is included in Fig 6B but this is not intuitive at first glance as it is the only bar in the graph that has no fosfomycin. It should be made clearer that this is the growth control.

Response: We thank the reviewer for this helpful comment. We agree that the control structure in Fig. 6B needed to be made clearer. The purpose of this assay was to compare the survival kinetics of representative *fos*-expressing constructs under a common fosfomycin exposure condition in the same RN4220 background, rather than to perform a strain-by-strain comparison of basal growth in the absence and presence of fosfomycin. We therefore included RN4220/pT without FOS as the untreated growth control and RN4220/pT + FOS as the FOS-exposed empty-vector control.
In the revised Fig. 6B, the RN4220/pT culture without FOS is now labelled as the untreated growth control, whereas RN4220/pT + FOS is labelled as the FOS-exposed empty-vector control. The legend further clarifies that all other groups in Fig. 6B were exposed to 16 mg/L FOS. We have also defined FOS as fosfomycin in the Fig. 6 legend to avoid confusion with *fos* gene names.

Reviewer #4:
I have nothing to add.

Response: We thank the reviewer for their assessment of the revised manuscript.

Reviewer #5:
I co-reviewed this manuscript with one of the reviewers who provided the listed reports. This is part of the Nature Communications initiative to facilitate training in peer review and to provide appropriate recognition for Early Career Researchers who co-review manuscripts.

Response: We thank the co-reviewer for their contribution to the review process.
